# Honey Bee Larval and Adult Microbiome Life Stages Are Effectively Decoupled with Vertical Transmission Overcoming Early Life Perturbations

**DOI:** 10.1128/mBio.02966-21

**Published:** 2021-12-21

**Authors:** Vienna Kowallik, Alexander S. Mikheyev

**Affiliations:** a Okinawa Institute of Science and Technology, Okinawa, Japan; b Australian National University, Canberra, ACT, Australia; University of Texas at Austin; CIML

**Keywords:** development, honey bee, host microbiome, metamorphoses, sociality

## Abstract

Microbiomes provide a range of benefits to their hosts which can lead to the coevolution of a joint ecological niche. However, holometabolous insects, some of the most successful organisms on Earth, occupy different niches throughout development, with larvae and adults being physiologically and morphologically highly distinct. Furthermore, transition between the stages usually involves the loss of the gut microbiome since the gut is remodeled during pupation. Most eusocial organisms appear to have evolved a workaround to this problem by sharing their communal microbiome across generations. However, whether this vertical microbiome transmission can overcome perturbations of the larval microbiome remains untested. Honey bees have a relatively simple, conserved, coevolved adult microbiome which is socially transmitted and affects many aspects of their biology. In contrast, larval microbiomes are more variable, with less clear roles. Here, we manipulated the gut microbiome of *in vitro*-reared larvae, and after pupation of the larvae, we inoculated the emerged bees with adult microbiome to test whether adult and larval microbiome stages may be coupled (e.g., through immune priming). Larval treatments differed in bacterial composition and abundance, depending on diet, which also drove larval gene expression. Nonetheless, adults converged on the typical core taxa and showed limited gene expression variation. This work demonstrates that honey bee adult and larval stages are effectively microbiologically decoupled, and the core adult microbiome is remarkably stable to early developmental perturbations. Combined with the transmission of the microbiome in early adulthood, this allows the formation of long-term host-microbiome associations.

## INTRODUCTION

Microbial symbionts (microbiomes) are often considered an essential part of the host phenotype, influencing important biological traits from nutrition to immunity to behavior (1–3). Hosts and their microbial symbionts can exert reciprocal selective effects on their ecological partners. For example, while the immune system plays an essential role in maintaining homeostasis with resident microbial communities, the resident bacteria also shape host immunity (4). Specifically, early time windows during development are important for setting host-microbiome trajectories in later life. For example, the order of species arrival can shape gut microbial composition (priority effect) (5, 6), and early disturbances can cause long-lasting changes to composition and function. The latter is specifically well studied in humans and mice with early life antibiotic-induced dysbiosis that then affects later microbial community composition which is correlated with health problems such as obesity (7–9). When host and microbiome interact over the course of many generations in stable or frequently occurring environments, they may undergo coordinated coadaptation to their shared environment and to each other (10). Such dynamics can be facilitated by vertical microbiome transmission, in contrast to horizontal acquisition of environmental microbes (11), and give rise to the evolution of specific functional roles of symbionts in the system (1, 12, 13). However, many barriers to vertical transmission exist, particularly associated with host reproduction and development (14). In many cases, elaborate methods evolved to inoculate otherwise largely sterile offspring with the maternal microbiome, either directly (e.g., during the birth or egg laying process) or indirectly using a shared environment as the vector (15, 16).

A special complication exists in holometabolous insects, where juvenile and adult stages are separated by metamorphosis. Holometabolism provides strategic advantages to species by avoiding intraspecific competition between larvae and adults and facilitating their adaptation to the different environments (the adaptive decoupling hypothesis) (17). While holometabolism is evolutionarily successful, it dramatically complicates the host-microbiome relationship because of the following. (i) Life stages have different ecological niches. (ii) Developmental reprogramming during pupation causes the gut and the microbes it contains to be purged. Microbial community stability across development varies widely across holometabolous insect taxa (18). Some insects evolved strategies to bypass metamorphosis-driven loss of early symbionts, such as developing structures in their guts to carry symbionts during pupation, or by inoculating environmental material to ensure reliable reinoculation after pupation (18). Here, sociality provides an efficient way to ensure the vertical transmission of a certain set of microbes. Insects that live in societies with overlapping generations can share the adult microbiome through trophallaxis and coprophagy (19, 20). This strategy appears to be effective, as many social insects have derived core adult microbiomes (18, 19). Such reliable reinoculation of newly emerged adults can make specific carryover strategies from larval to adult stage redundant.

However, symbionts do not necessarily need to be physically present to cause carryover effects. In several insects, early microbe-mediated immune priming in larvae affects the immune system during adulthood (21, 22), and host immunity together with the microbiome have been shown to jointly control opportunistic pathogens through development (21). As the immune system is key in selecting symbionts for colonization (4), early microbe-mediated changes to the immune system could influence colonization of beneficial symbionts in later life stages. Indeed, in some insect systems, early larval contact with specific microbes or pathogens affects later adult life history traits (23–25), and adult microbiome composition (26) demonstrating downstream effects. Whether larval symbionts can affect microbial associations in adult life is an understudied question (18). Specifically, the interplay between the microbiomes associated with the two distinct developmental stages and microbiome transmission remains poorly understood.

Here, we experimentally examined the effect of larval microbial composition on the adult microbiome in eusocial honey bees (Apis mellifera). Adult workers have a small, distinct, coevolved gut microbial community composed of ca. nine core bacterial phylotypes (27–31). This microbiome affects diverse traits of adult bees such as nutrition and immunity (32–36). In contrast to the adult microbiome, information on microbial composition, abundance, and function present in the digestive tract of larvae is conflicting and inconsistent (37–40). The larval microbiome is different and much more variable in comparison to the adult microbiome, although some taxa can be shared (41–45). There is some evidence of functional relationships between honey bee larvae and bacteria. Several bacterial and fungal pathogens specifically target larvae such as Paenibacillus larvae (foulbrood) (46). Some bacteria were shown to increase larval fitness (47–50), and diverse bacteria from larvae and the hive environment can inhibit larval pathogens (41, 49, 51–55). In addition, the larval immune system responds when it is inoculated with nonpathogenic bacteria (56), and responses to endogenous bacteria are species specific (57). These results indicate relationships between the early larval microbiome and the immune system of larvae. At the beginning and end of pupation, the exoskeleton, including large parts of the gut lining is shed or remodeled, eliminating internal bacteria from the larval stage (58, 59). During pupation, bacteria are largely absent, making pupae and newly emerged workers gnotobiotic (39). The adult gut microbiome is socially transmitted after emergence from pupation, offering the possibility of raising symbiont-depleted individuals in the lab. These can be reliably inoculated with a complete core microbiome by feeding bee hindguts (39).

In general, diverse carryover effects from larval stage to adult phenotypes exist in honey bees. For example, starvation stress in larvae affects various traits such as juvenile hormone titers causing adults to be more resilient toward starvation (60). Short-term hyperthermia in larval life increases the life span of adults and reduces sucrose responsiveness (61), and fungicide-containing food during larval development leads to a higher immune gene expression in emerging adults (62). From a host-microbiome point of view, the honey bee life stages are usually assumed to be decoupled, based on the gnotobiotic pupal stage (39). Indeed, while no direct carryover from larval to adult stage is known, whether the system is also indirectly decoupled (through, e.g., immune stimulation) remains to be tested.

Since their host-microbiome system is well characterized and experimentally tractable, honey bees are well suited for testing the hypothesis that larval and adult microbiome stages are decoupled. We used different diets and microbiome sources to experimentally manipulate larval microbiomes in the lab. We found that while microbiome and gene expression differences in larvae could be diverse, the adult microbiomes as well as gene expression profiles remained steady. This suggests a decoupling of the two microbiome stages and that vertical transmission can reliably overcome disturbances during early development.

## RESULTS

To create larvae with different microbial communities, we raised them in the lab and fed them with a royal jelly lab diet (63) without any addition (treatment C), with the addition of fresh bee bread (BB), adult gut inoculation (AG), larvae gut inoculation (LG), or addition of larvae gut and bee bread (LGBB). Larvae from the same frame raised in the hive were used as natural control (Hive). All treatments pupated in the lab, and emerging adults were exposed to the same adult gut microbiome pool. Survival and development rates and larva weight across treatments can be found in Fig. S1 in the supplemental material. We sequenced the gut bacterial community of 87 larval samples at different time points of their development (see Text S1), 53 adult bees (ca. three per cage = ca. nine per treatment) as well as the additional diet components and microbiome transfer pools. After sequencing the V3-V4 region of the bacterial 16S rRNA gene, run joining, read processing, and removing nonbacterial and rare sequences (<5 reads across sample set), the adult samples contained on average 101,440 reads (range, 39,675 to 142,622) and 279 amplicon sequence variants (ASVs). The larval samples contained on average, 71,656 reads (range, 7,474 to 152,820) and 1,436 ASVs. Rarefaction plots on the minimum sample counts (Fig. S2) showed that lines flattened quickly (<1,000 reads) in larval as well as adult samples, indicating sufficient depth.

10.1128/mBio.02966-21.1 TEXT S1 Text file with detailed methods containing the following parts: details of larva rearing protocol, larval diets and sampling details, DNA and RNA extraction details, 16S rRNA-based sequencing and community analyses, qPCR sequencing and analysis, RNA sequencing and analysis, and list of primers used in this study. Download Text S1, DOCX file, 0.06 MB. Copyright © 2021 Kowallik and Mikheyev. 2021 Kowallik and Mikheyev. https://creativecommons.org/licenses/by/4.0/ This content is distributed under the terms of the Creative Commons Attribution 4.0 International license.

**FIG 1:**
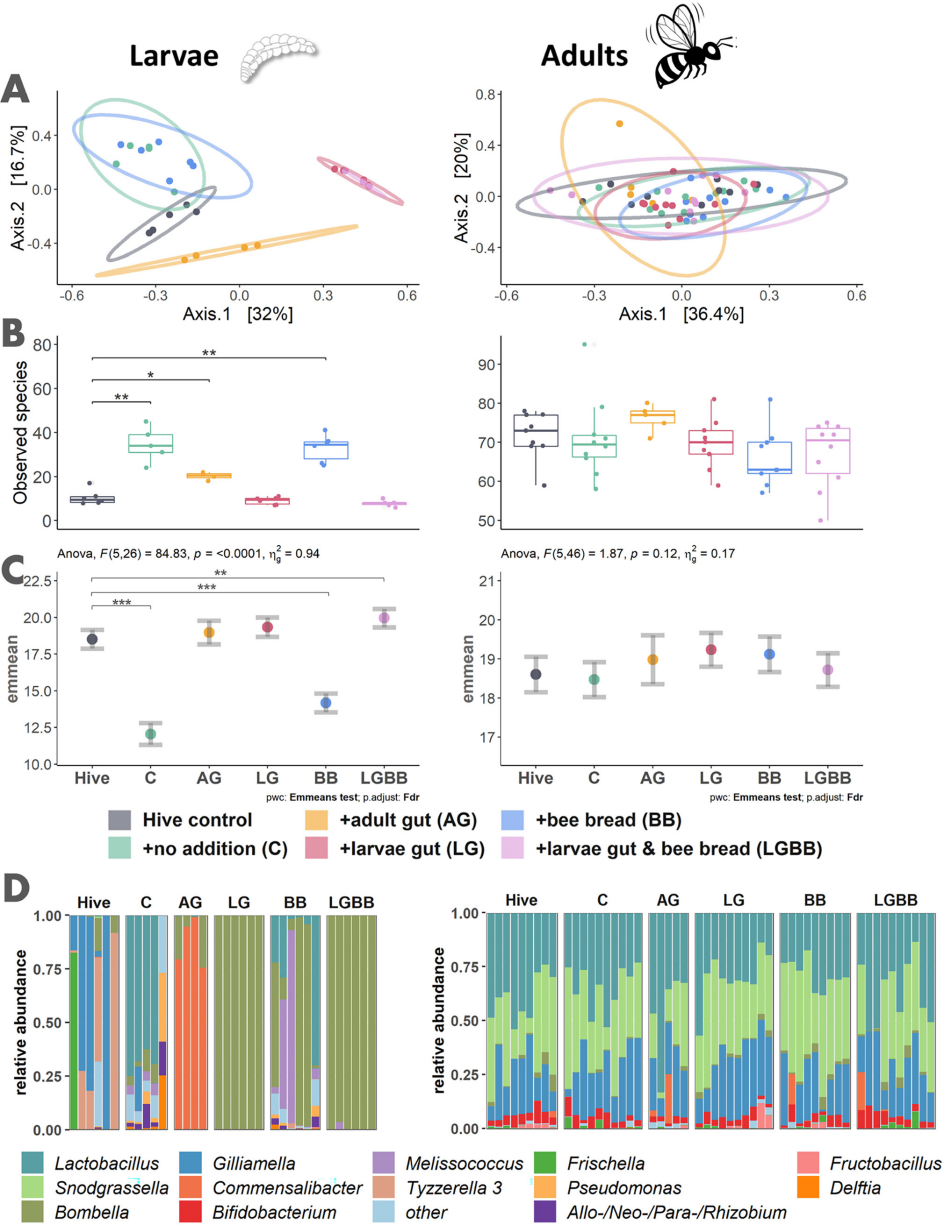
Larval gut microbial community and abundance are affected by rearing condition, but these differences are not mirrored in the adult microbiome. Alpha and beta diversity as well as taxonomy and total bacterial abundance show that composition and diversity of the late state larval microbiome on day six (left column) is strongly affected by rearing and diet conditions (see Fig. S5 for taxonomy of earlier time points), while the microbiome of adults that emerged from these treatments (right column) is not. The PCoA plot represents compositional differences between samples (beta diversity), separating the larval treatments, but not the adult samples (A). Ellipses represent 95% confidence intervals around treatment centroids. Pairwise FDR-corrected PERMANOVA verifies that all lab-treated larvae differ significantly from the hive control on day six (for C, *P* = 0.005, *F *= 4.6, and *R*^2^ = 0.3; for AG, *P* = 0.009, *F *= 7.5, and *R*^2^ = 0.5; for LG, *P* = 0.005, *F *= 10.7, and *R*^2^ = 0.5; for BB, *P* = 0.005, *F *= 4.5, and *R*^2^ = 0.3; for LGBB, *P* = 0.005, *F *= 11.3, and *R*^2^ = 0.5), while adult samples do not differ significantly (for C, *P* = 0.59, *F *= 0.9, and *R*^2^ = 0.05; for AG, *P* = 0.59, *F *= 1.2, and *R*^2^ = 0.09; for LG, *P* = 0.59, *F *= 0.9, and *R*^2^ = 0.05; for BB, *P* = 0.55, *F *= 1.8, and *R*^2^ = 0.1; for LGBB, *P* = 0.62, *F *= 0.7, and *R*^2^ = 0.04). Species richness (the number of observed species) varies between larval but not adult samples (B). Here, pairwise Wilcoxon rank sum tests, followed by FDR correction were used for statistical comparisons between treatments and hive control (***, *P* < 0.001; **, *P* < 0.01; *, *P* < 0.05) (for larvae, for C, *P* = 0.03 and *W* = 0; for AG, *P* = 0.05 and *W *= 0; for LG, *P* = 0.25 and *W *= 22; for BB, *P* = 0.03 and *W *= 0; for LGBB, *P* = 0.15 and *W *= 29.5) (for adults, for C, *P* = 0.41 and *W *= 55.5; for AG, *P* = 0.3 and *W *= 10.5; for LG, *P* = 0.41 and *W *= 50.5; for BB, *P* = 0.3 and *W *= 59; for LGBB, *P* = 0.33 and *W *= 61). See Fig. S2 for additional alpha diversity plots and all statistical details. 16S copy number abundance across treatments represented by estimated marginal means, including standard errors based on a one-way analysis of covariance (ANCOVA) model with treatment as s grouping factor, controlling for actin gene copy numbers (housekeeping gene) as covariate (C). Pairwise comparisons of estimated marginal means with FDR correction were used for statistical comparisons between treatments and hive control (for larvae, *P* < 0.001 for C, *P* = 0.37 for AG, *P* = 0.1 for LG, *P* < 0.001 for BB, and *P* = 0.005 for LGBB) (for adults, *P* = 0.71 for C, *P* = 0.53for AG, *P* = 0.25 for LG, *P* = 0.28 for BB, and *P* = 0.71 for LGBB). Taxonomy of bacterial genera with at least 1% relative abundance (everything else is combined in “other”) also shows taxonomic differences in larva (C). Across all these metrics, the larval gut microbiome is highly variable between treatments, but the adults were nonetheless colonized by the same core microbiome.

10.1128/mBio.02966-21.2 FIG S1 (A) Table with additional data on lab rearing and (B) figure on measured larval weight after pupation. (A) Survival and emergence data of lab rearing and pupation. Survival has not been monitored for the hive treatment. Larva survival excludes the samples taken on days 3 and 4. Treatments are hive reared control (Hive), royal jelly lab diet without any addition (treatment C), with the addition of fresh bee bread (BB), adult gut inoculation (AG), larval gut inoculation (LG) and addition of larvae gut and bee bread (LGBB). As visible in Fig. 3 of the main article, the addition of bee bread to the diet affected larval gene expression. The bee bread only treatment was the one with the fewest differentially expressed genes. In combination with the larva gut treatment, which alone showed most expression differences, it decreased the difference compared to the hive control. The adult gut treatment had intermediate numbers of differentially expressed genes which makes sense considering nurse bees consuming bee bread and therefore likely having residues inside the guts. Surprisingly, we observed the highest mortality during larval development in the bee bread treatment (B). As in comparison the C treatments despite low bacterial quantities, show the highest weight and survival from the lab treatments, the low overall bacterial yield in the BB treatment (Fig. 1) cannot be the reason for higher mortality. Potentially, the BB larvae that died were colonized by opportunistic pathogens and therefore selected against in our design. There is high relative abundance of *Melissococcus* reads present in some BB samples on day 6 (Fig. S6). A deeper look onto the species level reveals that it is in fact Melissococcus plutonius (a larval pathogen—European foulbrood). The lower percentage of successfully emerged pupae in the Hive control is likely caused by the process to pull the late-stage larvae out of the wax cells which added additional stress. (B) Larval weights 3 days after being put to pupation. Pairwise Wilcoxon rank sum tests between Hive treatment and all lab treatments with FDR correction show that the larvae naturally raised in the hive environment were heavier. Treatments are hive-reared control (Hive), royal jelly lab diet without any addition (treatment C), with addition of fresh bee bread (BB), adult gut inoculation (AG), larval gut inoculation (LG) and addition of larvae gut and bee bread (LGBB). Download FIG S1, PDF file, 1.1 MB. Copyright © 2021 Kowallik and Mikheyev. 2021 Kowallik and Mikheyev. https://creativecommons.org/licenses/by/4.0/ This content is distributed under the terms of the Creative Commons Attribution 4.0 International license.

10.1128/mBio.02966-21.3 FIG S2 Additional alpha diversity plots and statistics. For larvae, samples across the three sampling time points were used. Shannon index (A) accounts for abundance and evenness of ASVs in samples, while Observed species indicates the numbers of unique ASVs (B). Pairwise Wilcoxon rank sum tests, followed by FDR correction, were used for statistical comparisons between treatments and Hive control (***, *P* < 0.001; **, *P* < 0.01; *, *P* < 0.05); detailed statistics are reported in panel C. For adults, Shannon index of adult samples (D) and statistics derived from FDR-corrected pairwise Wilcoxon rank sum tests between hive control and lab treatments (E). Download FIG S2, PDF file, 2.4 MB. Copyright © 2021 Kowallik and Mikheyev. 2021 Kowallik and Mikheyev. https://creativecommons.org/licenses/by/4.0/ This content is distributed under the terms of the Creative Commons Attribution 4.0 International license.

### Larvae reared under different conditions develop different microbiomes.

Larvae reared under different conditions and diets developed strong differences in microbiome composition and abundance. The bacterial communities changed over developmental time and significantly differed between lab treatments and hive controls as shown in bacterial alpha diversity (Fig. S2), beta diversity (Fig. S4), as well as taxonomy (Fig. S5) and bacterial abundance (Fig. S6). Specifically, at the sixth day (before pupation initiation), treatments differed remarkably. Here, the multivariate homogeneity of groups is highly dispersed between treatments (PERMDISP, *P* = 0.01 and *F *= 3.97) (Fig. S3). This is supported by nonmetric multidimensional scaling (NMDS) (Fig. S4) and principal-coordinate analysis (PCoA) (Fig. 1A) based on Bray-Curtis dissimilarity matrices which consider presence/absence as well as abundance of ASVs. The ordination plots show distances between hive control and all lab-reared treatments, while the treatments receiving larva gut alone or in combination with bee bread are similar. Permutational multivariate analysis of variance (PERMANOVA) analysis on the distances shows that treatment significantly affects the microbiome at all three sampling time points (day 3, *P* = 0.001, *F *= 7.7, and *R*^2^ = 0.62), (day 4, *P* = 0.001, *F *= 5.7, and *R*^2^ = 0.7), (day 6, *P* = 0.001, *F *= 10, and *R*^2^ = 0.65). Pairwise PERMANOVA with Benjamini-Hochberg false discovery rate (FDR) corrections verifies that all treatments differ significantly from the hive control at day 6 (Fig. 1). In general, the microbiomes in larval treatments that received gut homogenate (AG, LG, and LGBB) were dominated by one or two bacterial genera. Therefore, they showed a low alpha diversity while the lab treatments that received only royal jelly diet (C) or bee bread as addition were colonized by a higher diversity (Fig. 1B). There was also a significant effect of treatment on total bacterial abundance as measured by quantitative PCR (qPCR) using analysis of covariance (ANCOVA) model with treatment as the grouping variable and controlling for copy numbers of the actin housekeeping gene as the covariate at day 3 [*F* (5, 23) = 34.2 and *P* < 0.001] as well as day 6 [*F* (5, 26) = 84.8 and *P* < 0.001]. Following pairwise comparisons of estimated marginal means (emmeans) with FDR correction showed on day 3 (Fig. S7) and 6 (Fig. 1C) significant differences indicating that larval treatments differed in both bacterial composition and abundance (Fig. 1D). Finally, RNA of individuals from days 3 and 6 were used as qPCR template to better represent the active bacterial community. Compared to DNA results of the same samples, the 16S copy numbers with RNA as the template were higher on day 3 (paired Wilcoxon signed-rank test, for Hive, *V* = 15 and *P* = 0.06; for C, *V *= 15 and *P* = 0.06; for BB, *V *= 15 and *P* = 0.06; for LG, *V *= 15 and *P* = 0.06) but lower on day 6 in all tested treatments (for Hive, *V *= 0 and *P* = 0.03; for C, *V *= 5 and *P* = 0.63; for BB, *V *= 0 and *P* = 0.03; for LG, *V *= 0 and *P* = 0.03) (Fig. S6).

10.1128/mBio.02966-21.4 FIG S3 Multivariate homogeneity of group dispersions (variances) on proportion data of the larval samples on day three (A) and day six (B). Multivariate tests for homogeneity of variance (with 999 permutations) revealed significant differences across treatments on day 3 (PERMDISP; *P* = 0.04 and *F *= 2.8) and day 6 (*P* = 0.01 and *F *= 3.97). Adult samples show homogeneous dispersion (C) (*P* = 0.53 and *F *= 0.85). Download FIG S3, PDF file, 0.9 MB. Copyright © 2021 Kowallik and Mikheyev. 2021 Kowallik and Mikheyev. https://creativecommons.org/licenses/by/4.0/ This content is distributed under the terms of the Creative Commons Attribution 4.0 International license.

10.1128/mBio.02966-21.5 FIG S4 Ordination plot of larval samples on day 6 (A) and statistical analysis (B) and adult samples (C) and statistical output (D). NMDS of Bray-Curtis dissimilarity which considers presence/absence as well as abundances of ASVs represents compositional differences between samples (beta diversity) are plotted. Ellipses represent 95% confidence intervals around treatment centroids, and the stress of the model is shown. Pairwise PERMANOVA tests on Bray-Curtis distances with 999 permutations using the ADONIS function were used. Treatments were tested against the respective Hive control and *P* value before and after FDR correction for multiple testing are shown. Significant differences are color marked. Download FIG S4, PDF file, 2.7 MB. Copyright © 2021 Kowallik and Mikheyev. 2021 Kowallik and Mikheyev. https://creativecommons.org/licenses/by/4.0/ This content is distributed under the terms of the Creative Commons Attribution 4.0 International license.

10.1128/mBio.02966-21.6 FIG S5 Taxonomic output of the sequenced larval samples. 24-year-old larvae on the start day of the experiment (A) as well as on day 3 (B), day 4 (C), and day 6 (D) during lab rearing were sampled. Relative abundance of bacterial taxa on genus level with an abundance of at least 5% across all larval samples are shown. All bacteria with a lower relative abundance are combined into “other.” The time points with legend next to it are shown individually here to make it easier to track the color taxon patterns for so many samples. Download FIG S5, PDF file, 1.8 MB. Copyright © 2021 Kowallik and Mikheyev. 2021 Kowallik and Mikheyev. https://creativecommons.org/licenses/by/4.0/ This content is distributed under the terms of the Creative Commons Attribution 4.0 International license.

10.1128/mBio.02966-21.7 FIG S6 Total bacterial abundance across treatments based on qPCR with DNA or RNA template. Log-transformed normalized bacterial abundance per sample of larval treatments on day 3 (A) and day 6 (B) as well as total normalized abundance of adults (C), normalized copy numbers of bacterial 16S per sample of DNA and RNA in larva samples at day 3 (D) and day 6 (E) and all abundance details (F). For panels A, B, and C for normalization, for each sample the raw 16S copy number per nanogram of DNA has been divided by the raw copy number of the actin housekeeping gene which has then been multiplied with dilution factor and DNA concentration in original sample to obtain the per sample value. These values have then been multiplied by the mean of actin gene copies across DNA samples for day 3 or day 6, respectively. For better visualization, log_10_ transformation of the larval samples are shown. Presented are data points, mean sand standard errors of the copy numbers per treatment. Pairwise Wilcoxon tests with following FDR correction on the total normalized abundance data were performed to compare the hive control with the treatments on day 3 (for C, *W *= 23 and *P* = 0.038; for AG, *W *= 25 and *P* = 0.013; for LG, *W *= 25 and *P* = 0.013; for BB, *W *= 21 and *P* = 0.1; for LGBB, *W *= 25 and *P* = 0.013), day 6 (for C, *W *= 0 and *P* = 0.007; for AG, *W *= 18 and *P* = 0.26; for LG, *W *= 31 and *P* = 0.05; for BB, *W *= 0 and *P* = 0.005; for LGBB, *W *= 36 and *P* = 0.005) and adults (for C, *W *= 41.5 and *P* = 0.81; for AG, *W *= 36 and *P* = 0.08; for LG, *W *= 74 and *P* = 0.09; for BB, *W *= 49 and *P* = 0.49; for LGBB, *W *= 41 and *P* = 0.81). For the DNA and RNA comparison in panels D and E, the raw 16S copy number per nanogram of DNA/RNA has been divided by the raw copy number of the actin housekeeping gene which has then been multiplied with dilution factor and DNA/RNA concentration in original sample to get a per sample estimate. These values have then been multiplied with the mean of actin gene copies across DNA samples or RNA samples, respectively. Presented are log-transformed data for easier visualization, means and standard errors of the copy numbers per larva are plotted. Paired Wilcoxon signed rank exact tests on the nontransformed 16S copy numbers per sample have been used to compare DNA and RNA from each treatment. (F) The table shows average, minimum, and maximum of total, normalized 16S copy numbers. Download FIG S6, PDF file, 2.8 MB. Copyright © 2021 Kowallik and Mikheyev. 2021 Kowallik and Mikheyev. https://creativecommons.org/licenses/by/4.0/ This content is distributed under the terms of the Creative Commons Attribution 4.0 International license.

10.1128/mBio.02966-21.8 FIG S7 16S copy number abundance across larval treatments on day 3. Estimated marginal means, including standard errors based on a one-way ANCOVA model with treatment as grouping factor and controlling for actin copy numbers (housekeeping gene) are plotted. Pairwise comparisons of the emmeans with Bonferroni correction were used for statistical comparisons between treatments and hive control (for C, *P* = 0.074; for AG, *P < *0.001; for LG, *P < *0.001; for BB, *P* = 0.074; for LGBB, *P* < 0.001). Download FIG S7, PDF file, 1.0 MB. Copyright © 2021 Kowallik and Mikheyev. 2021 Kowallik and Mikheyev. https://creativecommons.org/licenses/by/4.0/ This content is distributed under the terms of the Creative Commons Attribution 4.0 International license.

### Adult microbiome does not differ.

While the larvae showed strong differences, the adults did not differ in their established microbiomes. The core bacteria previously reported in adult bees colonized all treatments (Fig. 1D). PCoA (Fig. 1A) and NMDS (Fig. S4) show that the samples from all treatments cluster very closely. This is supported by FDR-corrected PERMANOVA with *P* values of >0.5 between any treatment and the Hive control (Fig. 1). In addition, treatments did not show significant effects on group dispersion (PERMDISP; *P* = 0.53 and *F *= 0.85) indicating homogeneous dispersion (Fig. S3). Deeper analysis on species and ASV (amplicon sequence variant) levels also showed no difference across treatments (Fig. S8). There was also no significant effect of treatment on total bacterial abundance [ANCOVA, *F* (5, 46) = 1.9 and *P* = 0.12]. Finally, pairwise comparisons with FDR correction of emmeans showed no significant differences between treatments and control (Fig. 1).

10.1128/mBio.02966-21.9 FIG S8 Analysis of microdiversity of core bacterial phylotypes in adult samples shows no treatment-specific strain selection. Reads were rarefied to even depth and only abundant species/ASV with a minimum of 1,000 reads in the data set are shown. Pairwise Wilcoxon rank sum tests were used for comparing treatments and Hive control (***, *P* < 0.001; **, *P* < 0.01; *, *P* < 0.05). (A) Abundance of different *Lactobacillus* species across treatments. Many reads could not be specified down to species level with the SILVA output and those not available (NA) are therefore excluded in panel A and further explained in panel B. (B) Exploring abundance of different ASVs belonging to *Lactobacillus* species with the SILVA output = “not_available” across the treatments. Blasting the ASV against the full NCBI Nucleotide collection revealed the following as the first full species hits: L. melliventris (ASV0220, ASV0391, ASV0547, ASV0578, ASV0842, ASV1173, ASV1240, and ASV1467), L. mellis (ASV0585, ASV0753, ASV0784, ASV1169, ASV1278, ASV2032, and ASV2253), L. mellifer (ASV1237), L. kunkeei (ASV0699), and L. kimbladii (ASV1002, ASV1534, and ASV1722). (C) Abundance of different ASV belonging to other core bacterial taxa for demonstration. Download FIG S8, PDF file, 0.9 MB. Copyright © 2021 Kowallik and Mikheyev. 2021 Kowallik and Mikheyev. https://creativecommons.org/licenses/by/4.0/ This content is distributed under the terms of the Creative Commons Attribution 4.0 International license.

### Adults show low levels of difference in gene expression, while larval differences are driven by diet.

RNA sequencing was performed to test for effects of diet/microbiome composition on larval and adult gene expression. Transcriptome sequencing (RNA-seq) reads per sample ranged from 6.3 to 21.0 million, with an average of 14.2 million reads per sample. After quality filtering and adapter trimming, an average of 63.4% (standard deviation [SD], 14.4%) of the reads per sample were pseudoaligned to generate transcript abundance for each annotated transcript in the recently updated honey bee genome annotation (Amel_HAv3.1).

**FIG 2:**
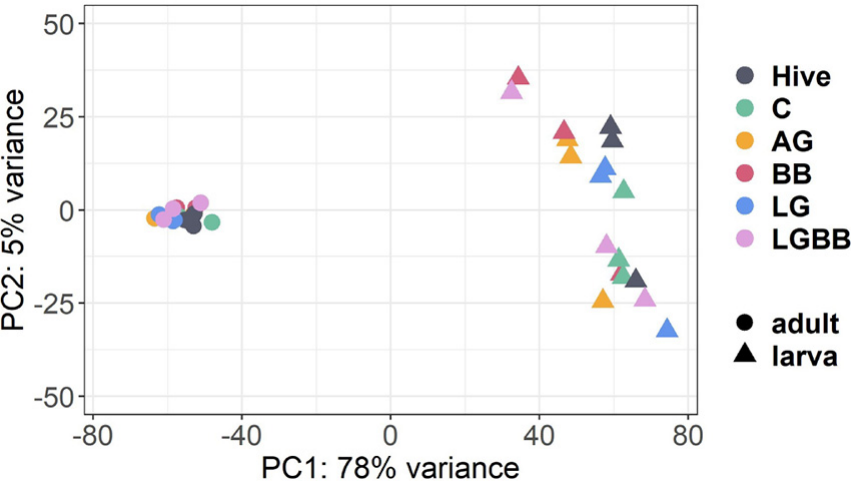
RNA expression across treatments and life stages. Principal-component analysis (PCA) on RNA data of adult and day 6 larval samples from the different treatments show clear separation in gene expression profiles between larval and adult samples. Specifically, high variation in gene expression between the larval samples is visible.

Gene expression profiles of the larval and adult samples show the same general pattern as the microbiome profiling. Adult samples cluster closely together, while larval samples show more variation between but also within treatments (Fig. 2).

Differential gene expression analysis was performed to characterize differences between lab treatments and hive controls in larvae and adults. Overall, there were few genes in adults significantly differently expressed after adjustment (Fig. S9). We found only four genes which were significantly different in more than one treatment. The uncharacterized gene LOC107965750 shows significantly higher expression in all lab treatments with the three gut transfer treatments being most different from the hive control (*P* = 0.045 for C, *P* < 0.001 for AG, *P* = 0.01 for BB, *P* < 0.001 for LG, and *P* < 0.001 for LGBB). The protein-coding gene calcium/calmodulin-dependent 3′,5′-cyclic nucleotide phosphodiesterase 1C, transcript variant X2 (LOC724389) shows higher expression in the gut transfer treatments (*P* = 0.01 for AG, *P* < 0.001 for LG, and *P* < 0.001 for LGBB), while the other two lab treatments do not differ significantly from the hive control (*P* = 1 for C and *P* = 0.25 for BB). The cuticle protein (LOC724464) is downregulated in the C treatment (*P* = 0.038) which is also the case for the pupal cuticle protein (LOC552685) in the C (*P* = 0.038) as well as AG treatment (*P* < 0.001). Exportin-6 (LOC726133), a nuclear export receptor specifically for profilin-actin complexes (64) is significantly upregulated in the LG (*P* = 0.048) and BB (*P* = 0.036) treatments. Hexamerin 110 (GeneID_551648), a storage protein during early honey bee development but also with a role in adults’ ovaries (65), shows significantly lower expression in the LGBB treatment (*P* = 0.006). The bee bread treatment with 14 differentially expressed genes is the most distinct treatment to the hive control. Here, the one downregulated gene (*P* = 0.038) maternal protein exuperantia, transcript variant X6 (LOC551582) is involved in reproductive processes, while the upregulated genes are involved in more general processes such as transcriptional (e.g., *P* = 0.036 for LOC726469 lysine-specific histone demethylase 1A) or transport (e.g., *P* = 0.036 for LOC409208 bumetanide-sensitive sodium-[potassium]-chloride cotransporter).

10.1128/mBio.02966-21.10 FIG S9 Overview of differentially expressed genes in the adult bee treatments. All genes which showed to be significantly different after *P* value adjustment between the hive control and any of the lab treatments were extracted and a heatmap of their expression across treatments is shown. It is additionally indicated per gene which of the lab treatment(s) differed significantly from the control. Download FIG S9, PDF file, 1.9 MB. Copyright © 2021 Kowallik and Mikheyev. 2021 Kowallik and Mikheyev. https://creativecommons.org/licenses/by/4.0/ This content is distributed under the terms of the Creative Commons Attribution 4.0 International license.

**FIG 3:**
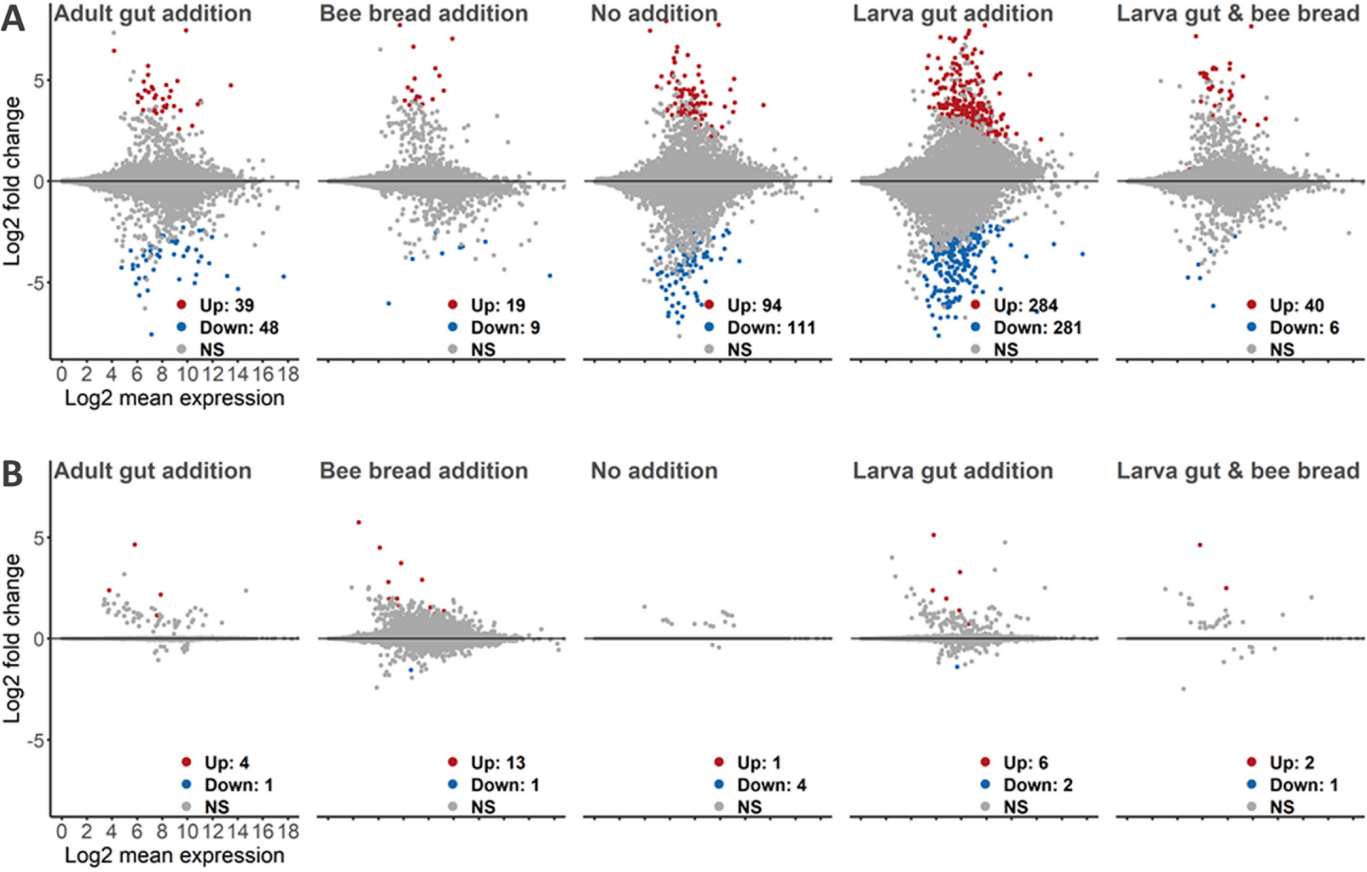
Expression of genes in larval (A) and adult (B) treatments against the respective hive control compared to MA plots. The *x* axis shows the average expression over the mean of normalized counts, and the *y* axis shows the gene-wise dispersion estimate’s shrunken log_2_ fold change. Red and blue points indicate significant up- or downregulation (FDR ≤ 0.05 determined by DESeq2) of individual genes. Larval differential gene expression was mainly influenced by diet components (e.g., bee bread addition seems to cause differences between lab and hive treatment to decrease), and the observed expression variations are not mirrored in the later adult stage. NS, not significant.

More expression variability but no clear pattern was seen in larval treatments compared to hive controls (Fig. 3) (significantly up- and downregulated genes and associated Gene Ontology (GO) terms for each treatment can be found in the GitHub repository [RNA folder]).

## DISCUSSION

Cotransmission of the host and microbiome via vertical transmission is thought to increase alignment of reproductive interests facilitating long-term cooperative interactions (66–68). The transition from larva to pupa in holometabolous insects poses a problem for cotransmission, and it is unclear whether the larval microbiome can affect the later adult microbiome (69). Eusociality, which is partially defined by overlap of generations, allows intimate microbiome transfer between individuals. This social transfer is hypothesized to have led to the evolution of distinctive and consistent gut communities with specialized functions found in social insects (70). We show the following. (i) The microbiome stages are effectively, also indirectly, decoupled between honey bee larva and adults. (ii) Vertical microbiome transfer after emergence overrides any variation in the larval microbiome in honey bees, allowing the colony to maintain a stable core adult microbiome even in the face of early perturbations.

### Early time windows during larval development are characterized by variation—the adult stage by consistency.

While the adult honey bee microbiome is well characterized, information about larvae is conflicting, which makes it difficult to understand the system holistically. Some studies barely detected bacteria in larvae, leading to the conclusion that any present bacteria represent transients and food contaminants rather than symbionts (37, 39). Other studies cultured diverse bacteria from larval guts (47, 53, 57), found almost equivalent bacterial copies per gram of gut material of fifth instar larvae and foragers (38) or could visualize dense bacterial presence in fifth instar larval guts of Apis cerana (55). Resident microbes differ per definition from transient microbes by their replication inside a host at a rate exceeding loss due to death or excretion (71, 72). Here, our qPCR results indicate that bacterial abundance in hive larvae was very low in the early development but increasing by ∼5,000-fold on day 6. Such an increase is expected considering the closed anatomy of the larval gut until the first and only defecation before the start of pupation (73). As DNA sequencing also captures dead bacteria which may accumulate in the closed guts, we additionally used RNA from 3- and 6-day-old larvae as the template for qPCR. Compared to DNA, RNA degrades more rapidly in the environment with an estimated half-life of a few minutes, which is why it is used as a method to identify active microbes (74). While we found higher numbers of 16S copies from RNA compared to the DNA template on day 3, this was opposite on day 6 across all treatments. This may indicate that, while there is an overall increase in bacteria over larva development, an accumulation of dead cells also occurs (see Fig. S6 in the supplemental material). Still, the high counts of RNA copies, indicate that a lot of bacteria are alive in the larval guts. However, alive does not mean growing, as for example, dormant cells will also contain RNA (75). Therefore, our data cannot differentiate whether bacterial cells are actively growing within the larval gut or whether bacterial cells are only accumulating.

While the core bacterial microbiome in emerged adults can be transferred by feeding macerated adult gut material (20), this method does not produce an adult-like microbiome in larvae, nor does the transfer of larval gut material create a microbiome similar to the hive-reared larva. In fact, none of our lab treatments resembled the hive control (Fig. 1). We observed compositional shifts of the microbiome during larval development especially in the hive control which coincides with other studies (40, 44) (Fig. S5). This indicates that the development time point of larvae, potential priority effects of symbiont colonization and maybe other factors only present in the hive environment (e.g., nurse contact) may be important for the selection and colonization of symbionts in larvae. It also suggests that in general lab-based studies on bee larval microbiome may be difficult to interpret, since the complexity of the hive environment cannot be mimicked. In contrast, controlling environmental effects is difficult in the hive, making it hard to disentangle the roles of different factors. As so far there is no option to rear microbiome-free honey bee larvae, we also cannot fully disentangle the effects from diet versus microbiome. While we see that gene expression changes in larvae are largely reset in young adults, this cannot be generalized for all genes under all conditions as shown in other studies that show gene expression in adults being affected by larval conditions (62, 76, 77).

While bacteria can be found in bee bread that also occur in larvae (78, 79), there is evidence that this habitat is in fact a rather poor one for bacterial growth (80). Our taxonomy and qPCR data also indicate that it does not seem to play an important role for microbiome establishment in larvae. Interestingly, it did seem to be a key factor affecting larval gene expression. The treatment receiving bee bread showed the smallest number of differentially expressed genes in comparison to the hive control (Fig. 3). The larval gut treatment was the most differentiated one, but in combination with bee bread, the differently expressed gene number decreased. Indeed, pollen associates such as phytochemicals and plant microRNAs have been shown to affect larval gene expression and caste development (81, 82).

Despite the strong variation in the larval stage, the well-described adult core microbiome colonized in all treatments and gene expression differences were limited. In general, the honey bee microbiome shows large variation on bacterial strain level (83), and recruitment there can be shaped by host genetics (84). While 16S sequencing has clear limitations when it comes to fine-scale taxonomic identification (85), the diversity of ASVs identified here verify microdiversity on bacterial strain level. This diversity was however found in all treatments with no clear pattern for treatment specificity (Fig. S8), confirming that there is also no deeper, hidden effects on adult microbial strain level. However, under hive conditions, a broader pool of surrounding environmental microbes in combination with natural social transmission which exposes the emerged bees to lower bacterial yields compared to the gut feeding method could potentially expose differences in microbiome composition, e.g., in susceptibility to becoming colonized by (opportunistic) pathogens. Also, early larval contact with specific coadapted pathogens could act as immune primers across life stages as shown in other insect systems such as beetles and social ants (23, 86).

Considering the massive difference in environment, social contact, diet, and microbiome in the artificial rearing compared to the controls as well as the physiological observed difference (all lab-treated larvae being lighter than the hive controls), finding only four genes that were different in more than one treatment seems small. However, three of these genes were differentially expressed in a manner that differentiated hive control bees from lab-reared bees. Therefore, some effects from the lab rearing affected gene expression in the adults. We cannot determine whether these effects are caused by the contact to different microbes, by differences in diet, or by the lack of natural stimuli which are provided in the hive environment.

While our lab rearing and microbiome transfer methods are not ecologically realistic, they provided the opportunity to control conditions, and have been widely used for this purpose by other studies (20, 36, 85). For instance, laboratory inoculation avoids potential biases in social interactions from the hive bees toward some or all introduced lab treatments, which could lead to altered microbiome states. In addition, using the same inoculation microbiome pool allowed testing for changes in relative abundances of taxa as well as strain level variation across the treatments. However, additional future experiments in a natural setting would be interesting.

### Two life stages and two host-microbiome strategies.

The evolution of sociality facilitated the development and maintenance of specialized, socially transmitted microbiomes in adult corbiculate bees. While larvae share the same environment and contact with adults, they do not seem to share the same microbiome transmission mode. The larval microbiome seems environmentally dependent, which has been observed in many other insects (70). Such environmental flexibility may be a source of adaptive potential (87). It does not mean complete random colonization, as the larval gut is a highly selective environment due to its low pH and antimicrobial peptides in royal jelly (88–90). The hive-reared bee larvae were heavier than all lab-treated bee larvae, but surprisingly, the one following closest was the C treatment (Fig. S1) which did not receive any microbe inoculum or bee bread and showed lowest overall bacterial density (Fig. 1). While in adults the microbiome is functionally involved in pollen digestion and host weight gain (35, 36, 91), microbes, at least the ones colonizing our lab treatments, do not seem to provide this function in larvae.

That being said, it is important to mention that not all organisms rely on symbionts for specialized functional purposes or well-being. Some even completely lack a resident microbiome which they avoid by creating a hostile gut environment, and in other cases, colonizing environmental microbes may act as only a protective barrier (71, 92). This scenario could be the case for honey bee larvae which would indicate that, if it does exist, a benefit from larval gut microbes would likely be measurable only when (opportunistic) pathogens are encountered. There is indication for such functional relationships between larvae and bacteria. Numerous studies demonstrated inhibition effects of various bacteria isolated from larvae, adults, or the hive environment on larval pathogens in culture media (41, 49, 51–55) and positive effects on the health of *in vitro*-reared larvae could also be confirmed (48–50). The ability of several larval bacteria to flourish in the antimicrobial royal jelly, indicating adaptation, is also interesting (40, 47). However, positive effects may also occur independent of any colonization inside the larval gut, e.g., just happening in the larval food or brood cell.

Further work is needed to identify and understand larva-microbiome relationships and a potential advantage of the environmentally flexible microbiome strategy. It may be worth mentioning that the queen microbiome is also characterized by high variability between colonies as well as being lower in bacterial abundance and lacking the typical adult worker core microbiome (93, 94) just as the larval microbiome is. This fact is interesting considering that both queens and larvae are fed with royal jelly by nurses.

### Vertical microbiome transmission allows decoupled life stages while maintaining a core coevolved microbiome.

While the decoupling of adult and larval microbiome stages in the honey bee system is assumed in literature, it was not completely proven so far as indirect effects were not explored. During pupation, individuals are gnotobiotic and also lack the ability to upregulate immune responses (95). Our data show that indeed the pupation resets the microbiome, allowing for colonization by the separate community of adult core bacteria. In general, having two decoupled microbiome stages may allow different functional microbiomes to exist in larval and adult developmental stages. Another advantage could be the avoidance of potential constraints in later host life derived from early microbiome selection in juvenile form (18). Our data support this theory. Moreover, reliable transmission of the adult microbiome allows combining the advantages from this adaptive microbiome decoupling with advantages accompanying vertical transmission of a coevolved microbiome. In general, vertical transmission reduces risks such as the loss of beneficial associations or colonization by opportunistic pathogens (70). Microbiome transfer between worker generations, as well as division of labor that keeps young workers inside the colony until their microbiome is fully developed, reduces opportunities for colonization by noncore microbial members (96). In addition, the microbiome mediates effects on the behavior, e.g., nestmate recognition cues are defined by colony-specific gut microbial communities (97). Such reciprocal effects between host behavior and microbiome which are highly manifested in many organisms, as demonstrated by the existence of a microbiota-gut-brain axis (98), are likely driving forces in the evolution of sociality and microbiome across the animal kingdom (99–101).

## MATERIALS AND METHODS

To test whether variation and dysbiosis in early colonizing larval microbiome may affect the establishment of a later adult microbiome, we raised honey bees through development and metamorphoses under controlled conditions in the lab. See Text S1 in the supplemental material for detailed methods and analysis.

### Larva rearing, treatments, and sampling.

In late September 2018, we grafted ∼24-h-old larvae from a single frame of an Apis mellifera colony in Okinawa, Japan. We marked this frame and left larvae of the same age for later sampling time points as hive-reared control individuals. For all steps in the standard larval rearing, we followed the protocol of Schmehl et al. (63).

**FIG 4:**
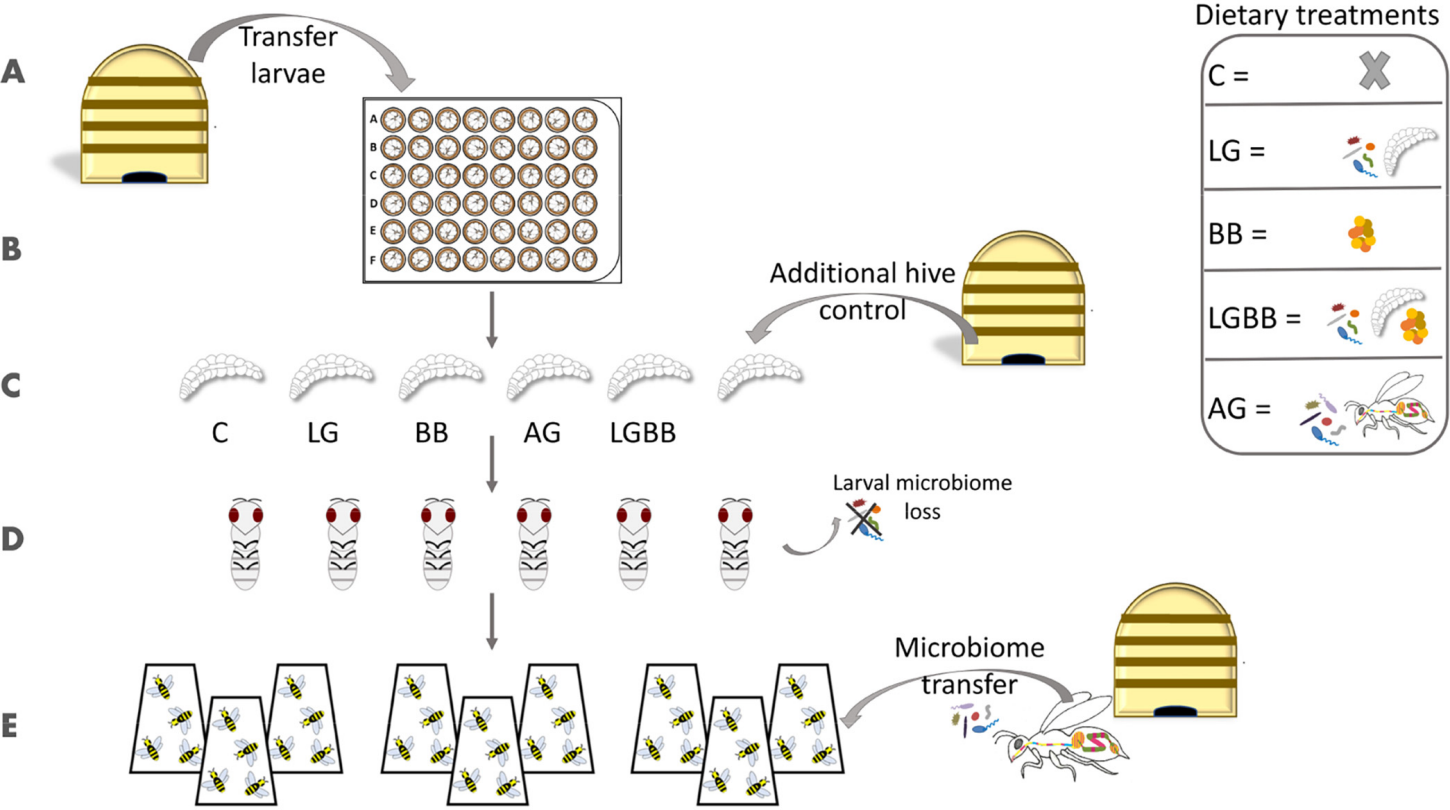
Experimental design. Larvae were grafted (A) and in the following 6 days lab reared with and without addition of gut microbiome pools and/or bee bread (B/C). On the sixth day, they were transferred to a new plate, and an additional hive control was taken (C). After pupation in the lab which naturally includes the loss of the larval microbiome during the morphological transformation process (D), emerged bees were distributed to three cages per treatment and a standardized adult microbiome pool was equally transferred to all cages (E).

To test whether early microbiome differences affect later microbiome establishment in adults, we had to create larvae that differ significantly in their microbiome composition without artificial disturbance by using chemicals, which would affect larval health. Therefore, we raised the larvae with five different diets and microbial components (Fig. 4) (48 individuals per treatment): (i) with standard royal jelly/sugar/yeast larval food (63) without addition (control [C]), (ii) adding larval gut microbiome (LG), (iii) adding bee bread (BB), (iv) adding larval gut and bee bread (LGBB), and (v) adding adult gut microbiome (AG) (details in Text S1). For adult gut transfer, we followed established protocols using the macerated hindguts of nurses (20). For the larval gut transfer, we macerated whole guts of late-stage larvae (shortly before defecating) as bacterial abundance is highest in this stage (38, 40). On the sixth rearing day, we moved the larvae for pupation. For having a comparable natural control, we pulled 35 larvae of the same age out of the marked frame and kept them under the same conditions in the pupation desiccator. To follow microbiome establishment, we snap-froze larvae at three time points after surface sterilization (Text S1).

### Adult maintenance in the lab.

From each treatment, we randomly distributed all emerging bees to three sterile cages to avoid batch effects within a time frame of 24 h. We excluded one cage of the AG treatment during the experiment after dropping it accidentally. In general, cages contained two prepared Eppendorf tubes as feeders to provide filter-sterilized 0.5 M sucrose solution and gamma-irradiated bee bread (30 kGy) *ad libitum*. To transfer adult microbiome, on days 1 and 2, 10 nurse bees from the same hive were surface sterilized and dissected. Hindguts were macerated in a 1:1 mix of phosphate-buffered saline (PBS) and 0.5 M sucrose, mixed with sterile bee bread, and equally distributed to all cages. During the experiment, food was replaced, and dead bees were removed on a daily basis. After 7 days to allow microbiome establishment (20), samples were taken, surface sterilized, and dissected, and whole guts as well as the whole abdomen were snap-frozen and stored at −80°C.

### Molecular methods. (i) Extractions, 16S rRNA sequencing, and analysis.

Adult guts and adult gut inoculum were extracted following the protocol in reference 27. For the inhibitor-rich larvae, we used the AllPrep PowerFecal DNA/RNA kit (Qiagen) following the manufacturer’s protocol. Library preparations and amplicon sequencing of the V3-V4 region of 16S rRNA region was performed by DNA Sequencing Section at Okinawa Institute of Science and Technology (OIST) on Illumina MiSeq v3 2 × 300-bp platform following the Illumina protocol. Reads were processed using QIIME2 version 2019.1 (102), denoising of the fastq files was performed using the denoise-paired command from the DADA2 software package (103), wrapped in QIIME2, including removal of chimeras using the “consensus” method. For taxonomic assignment, the QIIME2 q2-feature-classifier plugin (104) and the naive Bayes classifier (105), which we trained with our primers, were used on the SILVA release 132 (106, 107). Subsequent analyses for examining alpha and beta diversity as well as taxonomy on genus, species and ASV level were carried out in R, principally using the phyloseq package (108).

### (ii) qPCR sequencing and analysis.

For bacterial abundance, we amplified total copies of the 16S rRNA gene as well as the actin housekeeping gene to control for bias from extraction and sample size in 60 adult and 69 larval samples. We also amplified both target genes in the RNA from 43 larval samples to represent the more active bacterial community for direct comparison to the sample’s DNA. For cDNA synthesis from RNA, SuperScriptII reverse transcriptase (Invitrogen) was used according to the manufacturer’s protocol. 16S and actin target sequences were cloned in a pCRTM4-TOPO vector (Text S1) and amplified for standard curves. One-way ANCOVA was performed on log-transformed 16S copy numbers per sample as dependent and treatment as grouping variable while taking actin gene copies per sample as covariate into account. We performed pairwise comparisons between groups using the emmeans package (109) and plotted the obtained estimated marginal means. Finally, we calculated and plotted normalized absolute 16S copy numbers of DNA and RNA of samples.

### (iii) RNA sequencing and analysis.

For exploring gene expression profiles across different treatments, we sequenced mRNA from 18 adult samples (one bee for each experimental cage [two bees from one cage from the AG cages due to the cage loss]) and 18 day 6 larval samples (three per treatment). Novaseq reads were trimmed using AdapterRemoval (110) prior to being quantified using kallisto (111) with the honey bee transcriptome (version Amel_HAv3.1) as a reference, using default parameters. The R package DESeq2 was used to normalize and determine which genes were differentially expressed among treatments in adult as well as larval samples. Genes were considered differentially expressed between two treatments at an FDR-adjusted *P* value of <0.05. Gene Ontology (GO) enrichment analysis of the significantly differentially expressed genes between treatments were carried out using GOstats, GSEABase, and Category R packages (112).

### Availability of data and materials.

All data sets, including the lists of the significantly differentially expressed genes and GO terms for each treatment, codes to process sequences as well as code used in the analysis (in R markdown format) are available in the GitHub repository (https://github.com/kowallik/honey-bee-microbiome-development-larvae-adults).

Raw sequence data are available at DDBJ and NCBI under BioProject accession no. PRJDB12699, 16S sequences under Run accession no. DRR333007 to DRR333153, and RNA sequences under Run accession no. DRR333154 to DRR333191.
