# Supplementary material for: Honey Bee Larval and Adult Microbiome Life Stages Are Effectively Decoupled with Vertical Transmission Overcoming Early Life Perturbations
Source: mBio. 2021 Dec 21;12(6):e02966-21. doi: 10.1128/mBio.02966-21 (PMC8689520; doi:10.1128/mBio.02966-21)
Supplement: TEXT S1 [file mbio.02966-21-s0001.docx]

**Supplementary Information for**

**Honey bee larval and adult microbiome life stages are effectively decoupled with vertical transmission overcoming early life perturbations**

Vienna Kowallik^a^, Alexander S. Mikheyev^a,b^

^a^ Okinawa Institute of Science and Technology, 1919-1 Tancha Onna-son, 904-0495 Okinawa, Japan

^b^ Australian National University, Canberra, ACT, 2600, Australia

Email: vienna.kowallik@oist.jp, alexander.mikheyev@anu.edu.au

***Details of larvae rearing protocol***

All details of the larva rearing can be found in the detailed protocol of Schmehl et al. 2016 (1). Using sterile grafting tools, we placed larvae into 20 µl of larvae food mix in brown cell cups added to 48 well plates sterilized under UV light. The plates were placed in sterile desiccators with 400 ml K_2_SO_4_ salt solution in the bottom to boost the humidity to ~94%. Larvae were raised at 35 ̊C for six days. Larval food was prepared by mixing filter-sterilized water-yeast-sugar solution and royal jelly that was stored frozen at -20 ̊ C, and stored not longer than 48 hours in the fridge before warming it to 35 ̊C for feeding to larvae. On the sixth day, late-stage larvae were carefully placed onto sterile Kimwipe paper sheets inside new 48 well plates and left in a second desiccator at ~75% humidity (boosted with 400 ml NaCl) at 35 ̊C. Following this protocol, we are able to repeatedly raise healthy looking adult honey bees (although being slightly smaller than hive-reared bees) in the lab without signs of bacterial or fungal infections and without showing morphological signs of worker-queen intermediates.

***Larval diets and sampling details***

For transferring adult gut microbiome and larval gut microbiome to the lab reared larvae on lab day two and three, we collected five late-stage larvae from the same frame. They were processed immediately by surface sterilization in 80% ethanol for 1 minute with three following rinsing steps in sterile water, afterwards their guts were dissected. The guts were macerated in 300 ul PBS which got mixed with 5 ml larvae food preparation and fed to the LG treatment and LGBB treatment. The adult gut treatment was prepared exactly as the LG treatment, except for taking nurse bees and macerating their hindguts for transfer. On both days, we added 300 ul PBS solution to the diet of all other treatments to keep them comparable. For the bee bread addition, we collected and added fresh bee bread from the same hive on day three, four and five of the larvae rearing. Each day, we added 0.12 g of bee bread to 5 ml larvae food and fed this to the BB and LGBB treatments which gave us an overall bee bread concentration of ~2mg over each larval development which is reported to be the amount larva receive over development (2). All larvae treatments were reared for six days in the lab and checked daily to remove dead individuals and feed the alive ones with volumes according to the protocol. To follow microbiome establishment, larvae from all treatments including hive-reared larvae of the same age were sampled on several days always before feeding (see sampling schedule below). The sampled larvae were surface sterilized, in 80% ethanol for 1 minute with three following rinsing steps in sterile water with subsequent snap-freezing in liquid nitrogen and storage in the -80 freezer until extraction.

| **Sampling day (lab rearing)** | **treatment** | **sampled** |
| --- | --- | --- |
| Day 0 | Larvae ~24 hours old transferred to lab | 3 single larvae and 3 pools of 5 larvae |
| Day 3 | All plus hive control | 5 per treatment |
| Day 4 | All plus hive control | 3 per treatment |
| Day 6 | All plus hive control | ~5 per treatment (4 AG, 6 LG, 6 LGBB) |

***DNA and RNA Extractions details***

Adult guts as well as the gut inoculum for adults were extracted following the protocol in (3). This extraction protocol was not working for the larvae which are very inhibitor rich. Therefore, we used the AllPrep PowerFecal DNA/RNA Kit (Qiagen) following the manufacturers protocol on all larvae samples and their diet components (adult gut, larval gut and bee bread). The bead beating steps were performed using the Geno/Grinder® 2010. For both extraction methods, we ran two negative extraction controls, one in the middle and one in the final round of extractions to control for contamination happening in the process. RNA from surface-cleaned adult abdomen was extracted using TRIzol according to the manufacturer’s protocol. Total RNA quality and quantity were evaluated using the Bioanalyzer and Nanodrop. All extracts were kept in the -80°C freezer until further processing. Extracted DNA samples were quantified fluorometrically (Qubit, Thermo Fisher Scientific Inc.) and amplified with universal bacterial primers and run on 1% agarose gel to visualize successful extraction. For all negative extraction samples DNA quantity was non-detectable (see Github repository) and no gel band was obtained after PCR.

***16S rRNA–based sequencing and community analysis***

DNA of samples was submitted to DNA Sequencing Section at the Okinawa Institute of Science and Technology. Library preparation was performed based on Illumina protocol. Illumina barcoded primers (4) (see table below) were used to create a single amplicon of approximately 460 bp encompassing the V3-V4 region of bacterial 16S rRNA. 25-μl reactions were run for 25 cycles of amplification at an annealing temperature of 55 °C, using 2.5 µl of DNA template (5ng/uL) and the 2x KAPA HiFi HotStart ReadyMix (KAPA Biosystems, Inc) according to the manufacturer’s protocols. The PCR products were cleaned using AMpure XP beads (Beckman Colter, Inc) with 0.8× volume of beads to remove primer dimers. 50-μl reactions were run for 8 cycles of Index PCR amplification (see table below) at an annealing temperature of 55 °C, using 5 µl of PCR products, Nextera XT Index Kit v2, and the 2x KAPA HiFi HotStart ReadyMix according to manufacturer’s protocols. The Index PCR products were cleaned using AMpure XP beads with 1.12× volume of beads. Samples were pooled to equimolar concentration and sequenced on Illumina MiSeq v3 2 × 300 bp platform. Adult and larvae samples were run independently. Reads were demultiplexed on the basis of barcode sequences, allowing for one mismatch.

Reads were processed using QIIME2 version 2019.1 (5), denoising of the fastq files was performed using the denoise-paired command from the DADA2 software package (6), wrapped in QIIME2, including removal of chimeras using the “consensus” method. For both runs, primer sequences were removed by the DADA2 trim option and decreased sequence quality scores (< 20) at the end were truncated (trim-left-f = 17, trim-left-r = 21, trunc-len-f = 290, trunc-len-r 255). This resulted in a remaining overlap of ~85 bp in merged sequences. The result is an ASV table, a higher-resolution analog of the traditional OTU table. Both runs were joined then using the qiime feature-table merge function. For taxonomic assignment, the QIIME2 q2-feature-classifier plugin (7) and the Naïve Bayes classifier (8), which we trained with our primers, were used on the SILVA release 132 (9,10).

All following graphical and statistical comparisons were performed in R using the phyloseq package (11). Briefly, we first removed all non-bacterial sequences, mitochondrial and chloroplast sequences, and ASVs present in abundance <5 across samples from the datasets using *subset_taxa* and *prune_taxa* functions. We ran two negative extraction controls and a pure water sample together with the larval samples, we additionally ran Decontam in R which attempts to reduce potential contamination (which may occur more heavily in the low-bacteria yield larvae samples) with the Prevalence-based contaminant identification method using default parameters (12). However, this did not change anything for alpha- beta diversity and only filtered out the low-abundant genus “Delftia” from the >1% relative abundance taxonomic output. This genus may therefore potentially be a contaminant, however, in both sequenced negative extraction controls as well as the pure water sample, are also “Pseudomonas” and “Rhizobium” (which are both also abundant across larvae samples) present (figure in Github). Taken together, as the *Decontam* step did not apply strong changes, we could decide to present results without this step to treat adult and larval samples the same.

We plotted rarefaction curves of all samples using the ranacapa function *ggrare* (13) on the minimum sample depths. Alpha diversity of the rarefied samples was explored by plotting Observed species numbers and Shannon’s diversity index. Pairwise Wilcoxon rank sum tests with following FDR correction to correct for multiple testing were used to test for significant alpha diversity differences between treatments and respective hive controls. On proportion transformed data, NMDS and PCoA were performed on Bray-Curtis distances and plotted. To test for variation within groups, we used the *betadisper* function in the Vegan package, version 2.5-5 in R on the Bray-Curtis distance matrix on proportion data to calculate distances to group centroids. Subsequently, the output was plotted as ordination and boxplot for visualization and ANOVA was performed to test if one or more groups are more variable than the others. Multifactor PERMANOVA (14) on Bray-Curtis distances with 999 permutations using the ADONIS function with subsequent FDR corrections, were performed to test for microbiome compositional differences between treatments and respective hive controls. For taxonomic visualization we plotted relative abundances of genera across treatments and cycles. For a deeper characterization of the diversity of the adult core symbionts, we first extracted the abundant species for each Lactobacillus (>1000 reads) and plotted their abundances across treatments. We used online megablast against the full NCBI Nucleotide collection database on the ASV which did not give any information in the SILVA output (NAs). We also plotted the total abundance of ASVs of the other core phylotypes across treatments.

***qPCR sequencing and analysis.***

For bacterial abundance, we amplified total copies of the 16S rRNA gene as well as the housekeeping gene *Actin* to control for bias from extraction and sample’s size in 60 adult and 69 larval samples. We also amplified both target genes in the RNA of 43 larval samples to represent the more active bacterial community for direct comparison to the same sample’s DNA. For cDNA synthesis from RNA SuperScript™ II Reverse Transcriptase (Invitrogen™) was used according to manufacturer’s protocol.

We cloned the bacterial 16S and the housekeeping gene Actin sequences into a pCRTM4-TOPO vector for total bacterial quantification. For setting up 25 µL PCR products for plasmid construction, 0.125 µl EX-taq Polymerase (5U/µL) (TaKaRa), 2 µl dNTP, 1.25 µL of each 10 µm Primers together with 0.5 µL template cDNA and 2.5 µL 10xbuffer were used. PCR was carried out with the following conditions: initial denaturation at 98°C for 30 seconds, with 30 cycles of denaturation at 98°C for 10 seconds as well as 60°C for 30 seconds. Amplicons run on a 1% agarose gel were extracted using the Qiagen MinElute Gel Extraction Kit. The TOPO Cloning reaction was prepared with 1 µL of the purified PCR amplicon, 0.5 µL salt solution, 1 µL H2O and 0.5 µL of the TOPO vector. The mix was incubated at room temperature for 5 min, then transferred to ice. 1 µL was mixed with 25 µL TOP10 *E.coli*, incubated on ice for 10 min, then heated at 42C for 30 sec and again moved to ice for 2 min. Then 125 µL S.O.C. was added and the mix was rotated at 37C at 400 rpm for 1 h. Afterwards 125 µL was spread on an agar plate (+ampicillin & X-gal). Vectors from *E. coli* were purified using QIAprep Spin Miniprep Kit (QIAGEN).

Amplification was performed in house with the universal bacterial primers 27F and 355R (15) as well as the housekeeping gene primers Actin 1-F and Actin 1-R (see table below) (16) on a StepOnePlus (Applied Biosystems). Reactions (10 µl) were carried out in triplicate with 5 µl TB Green® Premix Ex Taq™ II (Takara), 0.4 µl 3 µM primer, 2 µl H_2_O, and 2 µl of 1 ng template DNA. The PCR cycle was 95°C (3 min) followed by 40 cycles of 95°C (5 s) and 60°C (60 s).

From the measured 16S copy number per ng, we calculated absolute copy number for the reaction templates then adjusted this based on dilution to calculate the total copy number for each sample. One-way Analysis of Covariance (ANCOVA) in R was performed after checking our data for model assumptions on the log-transformed 16S copy numbers per sample as dependent and treatment as grouping variable while taking *Actin* copy numbers per sample as covariate into account. Finally, we performed pairwise comparisons between treatment groups using the emmeans package (17) and plotted the obtained estimated marginal means across all treatments. We applied FDR corrections on the p-values comparing the Hive control to the other treatments. To get an idea about actual bacterial abundance per sample DNA or RNA we divided the raw 16S copy number by the *actin* copy number per sample for normalization (18). This value was multiplied by the dilution factors and DNA/RNA yield in the original extract to get a per sample estimate. Finally, this was multiplied with the mean raw *actin* copy number across samples of DNA or RNA (also age-specific mean was taken as larvae are different size). Paired Wilcoxon Signed Rank tests were performed to test for significant differences in total 16S copy numbers between RNA and DNA templates at day three and six.

***RNA sequencing and analysis***

# RNA library preparation and sequencing details (19)

***First strand cDNA synthesis***

To the RNA and spike-in mix, 2 µL of 10 µM poly T_START oligo5’-AATTGCAGTGGTATCAACGCAGAGCGGCCGCTTTTTTT-TTTTTTTTTTTTTTTTTTTTTTVN were added. The 9 µL mixture was incubated at 65°C for 3 minutes, and then chilled on ice. The 11 µL reaction mixture containing 4 µL of 5x first strand synthesis buffer (Invitrogen), 1 µL of 10 mM dNTP (Promega), 2 µL of 0.1 M DTT (Invitrogen), 2 µL of 12 µM template switching RNA oligo (5’-AAGCAGUGGUAUCAACGCAGAGUACAUGGG), 1 µL RNase inhibitor (Qiagen) and 1 µL superscript II reverse transcriptase (Invitrogen) were added to each sample. The reactions were incubated at 42°C for 60 minutes and the enzyme was heat inactivated at 65°C for 15 minutes. 80 µL MilliQ of water was added to each cDNA reaction.

***Second strand cDNA synthesis***

Second strand cDNA was synthesized with a limited number of PCR cycles. The 50 µL PCR reaction consisted of 1x Phusion HF buffer (Thermo Scientific), 200 µM dNTP (Promega), 0.5 µM START primer (5’-CGCCAGGGTTTTCCCAGTCACGACAATTGCAGTGGTATCAACGCAGA), 0.5 µM TS_long primer (5’-CTTGTAGGTTAAGTGGAGAGCTAACAATTT-CACACAGGAAAGCAGTGGTATCAACGC), 0.5 µL of 2 U/ µL Phusion DNA polymerase (Thermo Scientific) and 10 µL diluted cDNA. 50 µL PCR reactions were set up for each cDNA sample. PCR was carried out with the following conditions: initial denaturation at 98°C for 30 seconds, with 20 cycles of denaturation at 98°C for 10 seconds, 68°C for 6 min, followed by final extension at 72° C for 10 min. PCR products were purified by solid phase reversible immobilization using Dynabeads MyOne Carboxylic Acid (Invitrogen), 17% PEG was used for purification. The concentration of the DNA was measured with Quant-iT PicoGreen dsDNA Assay Kit (Invitrogen).

***Nextera XT library preparation and sequencing***

One ng of ds-cDNA was used for library preparation using the Nextera XT DNA sample kit (Illumina) according to manufacturer’s directions following the protocol of Aird et. al (20). Twelve cycles of PCR were used for library amplification. The libraries were size selected before sequencing. In the first selection step, 100 µL of 13% PEG-6000/NaCl/Tris and 10 µL prepared Dynabeads were added to the library and resuspended. The mixture was incubated for 5 minutes. The tube was then placed on a magnetic stand for 5 minutes. 150 µL supernatant were transferred to a new tube while the beads were discarded. In the second selection, 100 µL of 13.5% PEG-6000/NaCl/Tris and 10 µL prepared Dynabeads were added to the supernatant and mixed. The mixture was incubated for 5 minutes followed by bead separation on the magnetic stand. This time, the supernatant was discarded, and the beads were saved. The beads were washed twice with 70% ethanol (with 10 mM Tris, pH 6) and dried for 5 minutes. The tubes were then taken off the magnetic stand and DNA was eluted from the beads by resuspending them in 15 µL EB. After 5 minutes incubation, beads were separated from the DNA solution on the magnetic stand. The eluant contained the purified library with peak size of around 380bp. The libraries were analyzed with Bioanalyzer High Sensitivity DNA Kit (Agilent Technologies).

The quantity of the library was estimated by Quant-iT PicoGreen dsDNA Assay Kit and equimolar libraries were pooled. Quantitative PCR (KAPA Biosystems) was used to estimate the concentration of the libraries. The pooled library was sequenced using pair-end strategy (read length 50bp) on Novaseq 6000 platform at OIST.

***RNA sequence analysis***

For exploring gene expression profiles across different treatments, we did mRNA sequencing of 18 adult and 18 day six larval samples (three per treatment). RNA library preparation was done with the Illumina Nextera-XT DNA sample Prep kit according to the manufacturer’s protocol. The pooled libraries were sequenced using pair-end strategy (read length 50bp) on Novaseq 6000 platform at OIST. Reads were trimmed using AdapterRemoval (21) (–trimwindows 5 –minquality 20) to remove adapter sequences and low quality reads prior to being quantified using kallisto (22) with the honey bee transcriptome (version Amel_HAv3.1) as a reference, using default parameters.

The R package DESeq2 was used to normalize data and determine which genes were differentially expressed among treatments in adult as well as larval samples (23). We obtained the gene model from the Ensemble BioMart database (data set 20 of metazoan ensemble; *Apis mellifera* (DH4) genes (Amel_HAv3.1)) by using makeTxDbFromBiomart function from the GenomicFeatures package (24) as well as metadata for these transcripts using the biomart R package (25). To reduce the variability of lowly expressed genes shrunken log2 fold changes (LFC) were created by the *lfcShrink* function with type “apeglm”. A principal component analysis (PCA) was performed on varianceStabilizingTransformation (vst) transformed data of all samples, and for better visualization adult samples only, to evaluate the distribution according to expression profiles. All treatments were compared against the hive control and to explore the effect of diet components onto larva expression into more detail, we repeated the analysis with the no-addition (C) lab treatment as comparison basis. Genes were considered to be differentially expressed between two treatments at an FDR adjusted *p*-value <0.05. MA plots for adult and larva samples by the ggpubr *ggmaplot* function were created to visualize differences between lab treatments and the respective hive controls by plotting shrunken LFCs for each gene against its average expression. Gene ontology (GO) enrichment analysis of the significantly differentially expressed genes between treatments were carried out using GOstats, GSEABase and Category R packages (26).

**List of primers used in this study**

| **Primer** | **Sequence** | **Reference** |
| --- | --- | --- |
| Forward Illumina barcoded Primer | 5'TCGTCGGCAGCGTCAGATGTGTATAAGAGACAGCCTACGGGNGGCWGCAG-3’ | (4) |
| Reverse Illumina barcoded Primer | 5’GTCTCGTGGGCTCGGAGATGTGTATAAGAGACAGGACTACHVGGGTATCTAATCC-3’ | (4) |
| Index 1 Primer | 5’-CAAGCAGAAGACGGCATACGAGAT[i7bases]GTCTCGTGGGCTCGG 3' |  |
| Index 2 Primer | 5’AATGATACGGCGACCACCGAGATCTACAC[i5bases]TCGTCGGCAGCGTC 3' |  |
| Universal bacterial 27F Primer | 5’-AGAGTTTGATCCTGGCTCAG-3’ | (15) |
| Universal bacterial 355R Primer | 5’-CTGCTGCCTCCCGTAGGAGT-3’ | (15) |
| Actin 1-F | 5’-AATCACTGCCCTAGCACCAT-3’ | (16) |
| Actin 1-R | 5’-GGAAGGTGGACAAAGAAGCA-3’ | (16) |

**References**

1. Schmehl DR, Tomé HVV, Mortensen AN, Martins GF, Ellis JD. Protocol for the in vitro rearing of honey bee (Apis mellifera L.) workers. Journal of Apicultural Research. 2016 März;55(2):113–29.

2. Babendreier D, Kalberer N, Romeis J, Fluri P, Bigler F. Pollen consumption in honey bee larvae: a step forward in the risk assessment of transgenic plants. Apidologie. 2004 May 1;35(3):293–300.

3. Engel P, James RR, Koga R, Kwong WK, McFrederick QS, Moran NA. Standard methods for research on Apis mellifera gut symbionts. Journal of Apicultural Research. 2013;52(4):1–24.

4. Klindworth A, Pruesse E, Schweer T, Peplies J, Quast C, Horn M, et al. Evaluation of general 16S ribosomal RNA gene PCR primers for classical and next-generation sequencing-based diversity studies. Nucleic Acids Res. 2013 Jan;41(1):e1.

5. Bolyen E, Rideout JR, Dillon MR, Bokulich NA, Abnet CC, Al-Ghalith GA, et al. Reproducible, interactive, scalable and extensible microbiome data science using QIIME 2. Nat Biotechnol. 2019 Aug;37(8):852–7.

6. Callahan BJ, McMurdie PJ, Rosen MJ, Han AW, Johnson AJA, Holmes SP. DADA2: High-resolution sample inference from Illumina amplicon data. Nature Methods. 2016 Jul;13(7):581–3.

7. Bokulich NA, Kaehler BD, Rideout JR, Dillon M, Bolyen E, Knight R, et al. Optimizing taxonomic classification of marker-gene amplicon sequences with QIIME 2’s q2-feature-classifier plugin. Microbiome. 2018 May 17;6(1):90.

8. Wang Q, Garrity GM, Tiedje JM, Cole JR. Naïve Bayesian Classifier for Rapid Assignment of rRNA Sequences into the New Bacterial Taxonomy. Appl Environ Microbiol. 2007 Aug;73(16):5261–7.

9. Quast C, Pruesse E, Yilmaz P, Gerken J, Schweer T, Yarza P, et al. The SILVA ribosomal RNA gene database project: improved data processing and web-based tools. Nucleic Acids Res. 2013 Jan 1;41(D1):D590–6.

10. Yilmaz P, Parfrey LW, Yarza P, Gerken J, Pruesse E, Quast C, et al. The SILVA and “All-species Living Tree Project (LTP)” taxonomic frameworks. Nucleic Acids Res. 2014 Jan 1;42(D1):D643–8.

11. McMurdie PJ, Holmes S. phyloseq: An R Package for Reproducible Interactive Analysis and Graphics of Microbiome Census Data. PLOS ONE. 2013 Apr 22;8(4):e61217.

12. Davis NM, Proctor DM, Holmes SP, Relman DA, Callahan BJ. Simple statistical identification and removal of contaminant sequences in marker-gene and metagenomics data. Microbiome. 2018 Dec 17;6(1):226.

13. Kandlikar GS, Gold ZJ, Cowen MC, Meyer RS, Freise AC, Kraft NJ, et al. ranacapa: An R package and Shiny web app to explore environmental DNA data with exploratory statistics and interactive visualizations. F1000Research. 2018;7.

14. Anderson MJ. A new method for non-parametric multivariate analysis of variance. Austral Ecology. 2001;26(1):32–46.

15. Lane D. 16S/23S rRNA sequencing. Nucleic acid techniques in bacterial systematics. 1991;115–75.

16. Velasque M, Qiu L, Mikheyev AS. The Doublesex sex determination pathway regulates reproductive division of labor in honey bees. bioRxiv. 2018 May 4;314492.

17. Lenth R. emmeans: Estimated Marginal Means, aka Least-Squares Means. R package version 1.5. 4. 2021;

18. Kešnerová L, Emery O, Troilo M, Liberti J, Erkosar B, Engel P. Gut microbiota structure differs between honeybees in winter and summer. ISME J. 2020 Mar;14(3):801–14.

19. Tin MMY, Rheindt FE, Cros E, Mikheyev AS. Degenerate adaptor sequences for detecting PCR duplicates in reduced representation sequencing data improve genotype calling accuracy. Mol Ecol Resour. 2015 Mar;15(2):329–36.

20. Aird SD, Watanabe Y, Villar-Briones A, Roy MC, Terada K, Mikheyev AS. Quantitative high-throughput profiling of snake venom gland transcriptomes and proteomes (Ovophis okinavensis and Protobothrops flavoviridis). BMC Genomics. 2013 Nov 14;14(1):790.

21. Schubert M, Lindgreen S, Orlando L. AdapterRemoval v2: rapid adapter trimming, identification, and read merging. BMC Research Notes. 2016 Feb 12;9(1):88.

22. Bray NL, Pimentel H, Melsted P, Pachter L. Near-optimal probabilistic RNA-seq quantification. Nat Biotechnol. 2016;34(5):525–7.

23. Love MI, Huber W, Anders S. Moderated estimation of fold change and dispersion for RNA-seq data with DESeq2. Genome Biology. 2014 Dec 5;15(12):550.

24. Lawrence M, Huber W, Pagès H, Aboyoun P, Carlson M, Gentleman R, et al. Software for Computing and Annotating Genomic Ranges. PLOS Computational Biology. 2013 Aug 8;9(8):e1003118.

25. Durinck S, Moreau Y, Kasprzyk A, Davis S, De Moor B, Brazma A, et al. BioMart and Bioconductor: a powerful link between biological databases and microarray data analysis. Bioinformatics. 2005 Aug 15;21(16):3439–40.

26. Falcon S, Gentleman R. Using GOstats to test gene lists for GO term association. Bioinformatics. 2007 Jan 15;23(2):257–8.
