# Supplementary material for: Honey Bee Larval and Adult Microbiome Life Stages Are Effectively Decoupled with Vertical Transmission Overcoming Early Life Perturbations
Source: mBio. 2021 Dec 21;12(6):e02966-21. doi: 10.1128/mBio.02966-21 (PMC8689520; doi:10.1128/mBio.02966-21)

A

treatment	start number	sampled	died	larvae survival	survived	put to pupation	emerged	emerged
Hive	/	14	/	/	/	32	23	72%
AG	48	14	10	30	0.63%	24	20	83%
BB	48	14	18	22	0.46%	16	15	94%
C	48	14	5	35	0.73%	29	25	86%
LG	48	14	6	34	0.71%	28	27	96%
LGBB	48	14	10	30	0.63%	24	20	83%

B

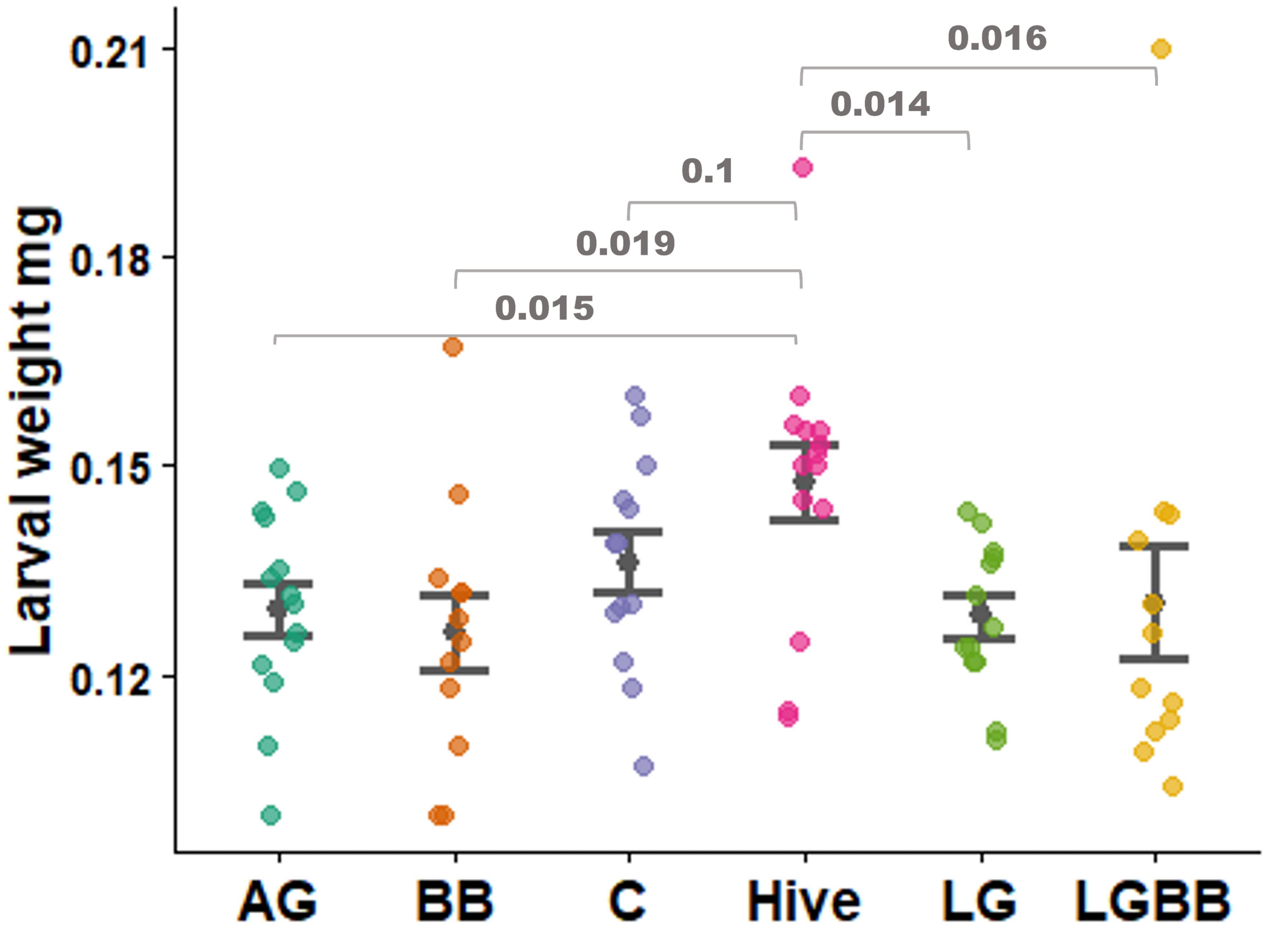

Supplement: FIG S1 [file mbio.02966-21-sf001.pdf]
