# Supplementary material for: Honey Bee Larval and Adult Microbiome Life Stages Are Effectively Decoupled with Vertical Transmission Overcoming Early Life Perturbations
Source: mBio. 2021 Dec 21;12(6):e02966-21. doi: 10.1128/mBio.02966-21 (PMC8689520; doi:10.1128/mBio.02966-21)
Supplement: FIG S2 [file mbio.02966-21-sf002.pdf]

Larvae

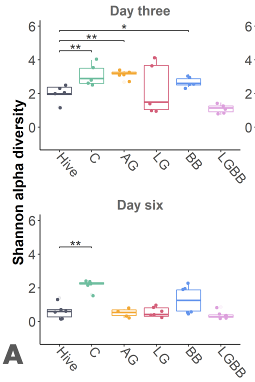

A

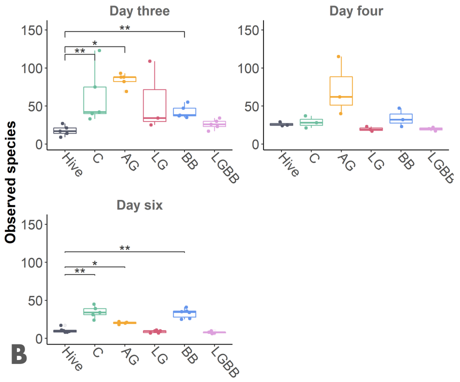

B

treatment	time point	Pairwise-Wilcoxon statistics
C	day three	Shannon: $p=0.02$, $W=0$; Observed: $p=0.02$, $W=0$
AG	day three	Shannon: $p=0.02$, $W=0$; Observed: $p=0.02$, $W=0$
LG	day three	Shannon: $p=0.84$, $W=14$; Observed: $p=0.02$, $W=1$
BB	day three	Shannon: $p=0.05$, $W=2$; Observed: $p=0.02$, $W=0$
LGBB	day three	Shannon: $p=0.07$, $W=22$; Observed: $p=0.12$, $W=4.5$
C	day four	Shannon: $p=0.5$, $W=2$; Observed: $p=1$, $W=4$
AG	day four	Shannon: $p=1$, $W=5$; Observed: $p=0.17$, $W=0$
LG	day four	Shannon: $p=0.25$, $W=9$; Observed: $p=0.17$, $W=9$
BB	day four	Shannon: $p=0.5$, $W=2$; Observed: $p=0.88$, $W=3$
LGBB	day four	Shannon: $p=0.25$, $W=9$; Observed: $p=0.17$, $W=9$
C	day six	Shannon: $p=0.02$, $W=0$; Observed: $p=0.025$, $W=0$
AG	day six	Shannon: $p=1$, $W=11$; Observed: $p=0.05$, $W=0$
LG	day six	Shannon: $p=0.94$, $W=17$; Observed: $p=0.25$, $W=22$
BB	day six	Shannon: $p=0.45$, $W=9$; Observed: $p=0.025$, $W=0$
LGBB	day six	Shannon: $p=0.94$, $W=21$; Observed: $p=0.15$, $W=29.5$

C

Adults

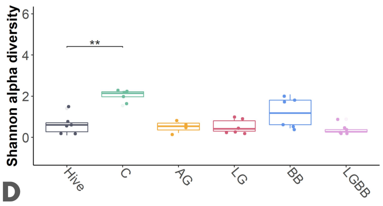

D

treatment	Pairwise-Wilcoxon statistics
C	Shannon: $p = 0.35$, W = 61 ; Observed: $p = 0.41$, $W = 55.5$
AG	Shannon: $p = 0.28$, $W = 10$; Observed: $p = 0.3$, $W = 10.5$
LG	Shannon: $p = 0.43$, $W = 52$; Observed: $p = 0.41$, $W = 50.5$
BB	Shannon: $p = 0.04$, $W = 70$; Observed: $p = 0.3$, $W = 59$
LGBB	Shannon: $p = 0.72$, $W = 50$; Observed: $p = 0.33$, $W = 61$

E
