# Supplementary figures and images for: Honey Bee Larval and Adult Microbiome Life Stages Are Effectively Decoupled with Vertical Transmission Overcoming Early Life Perturbations
Source: mBio. 2021 Dec 21;12(6):e02966-21. doi: 10.1128/mBio.02966-21 (PMC8689520; doi:10.1128/mBio.02966-21)

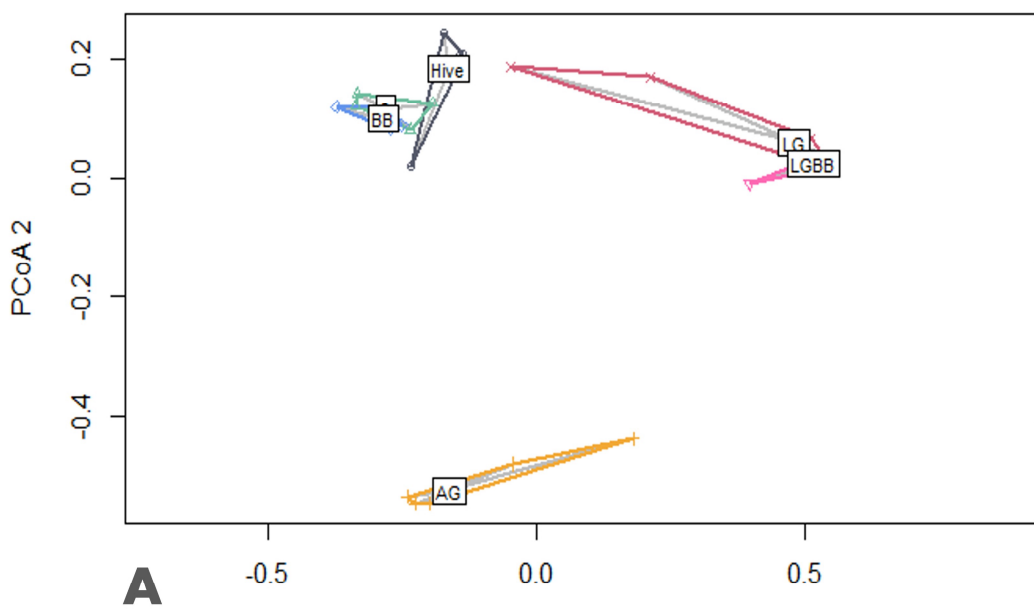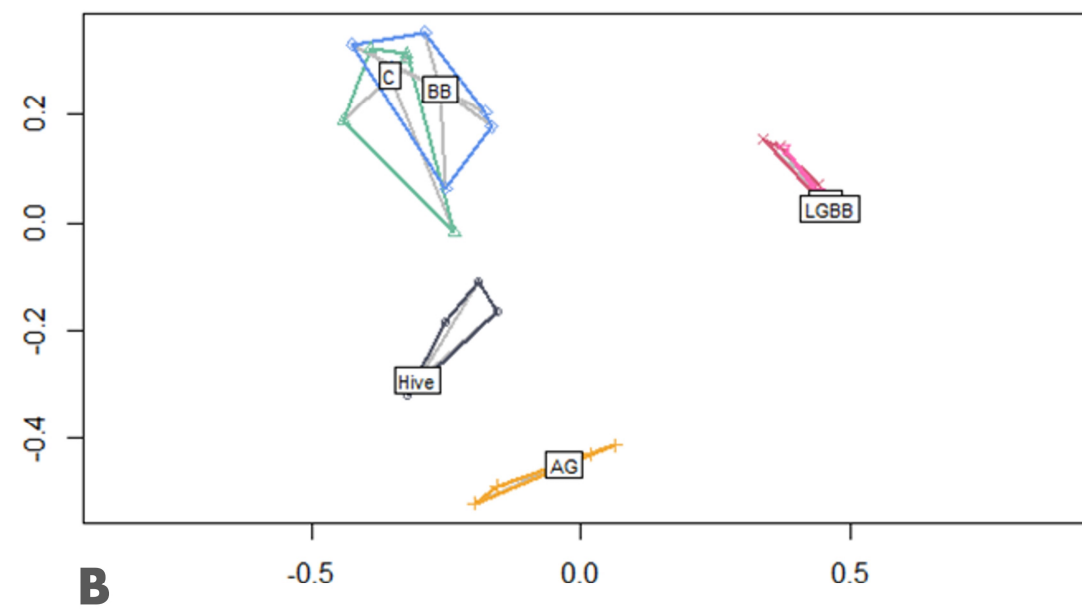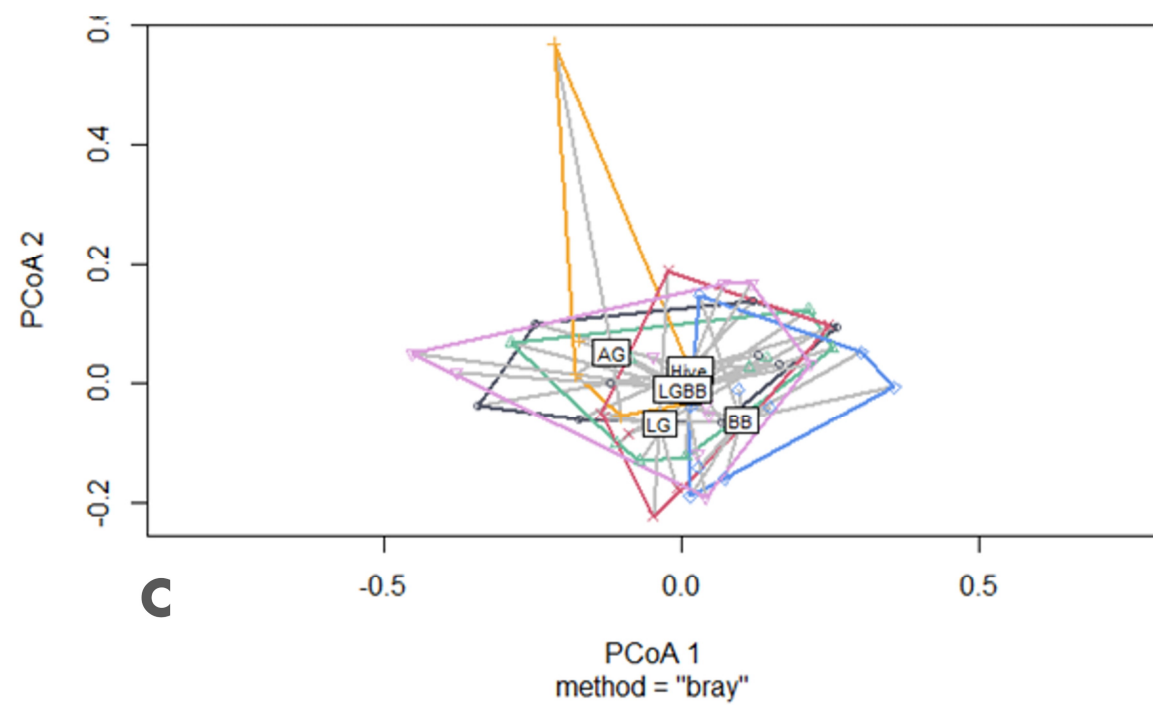

Supplement: FIG S3 [file mbio.02966-21-sf003.pdf]

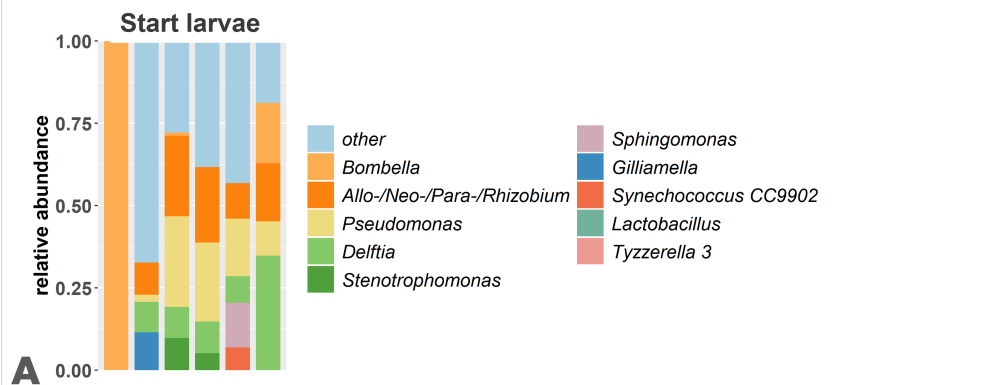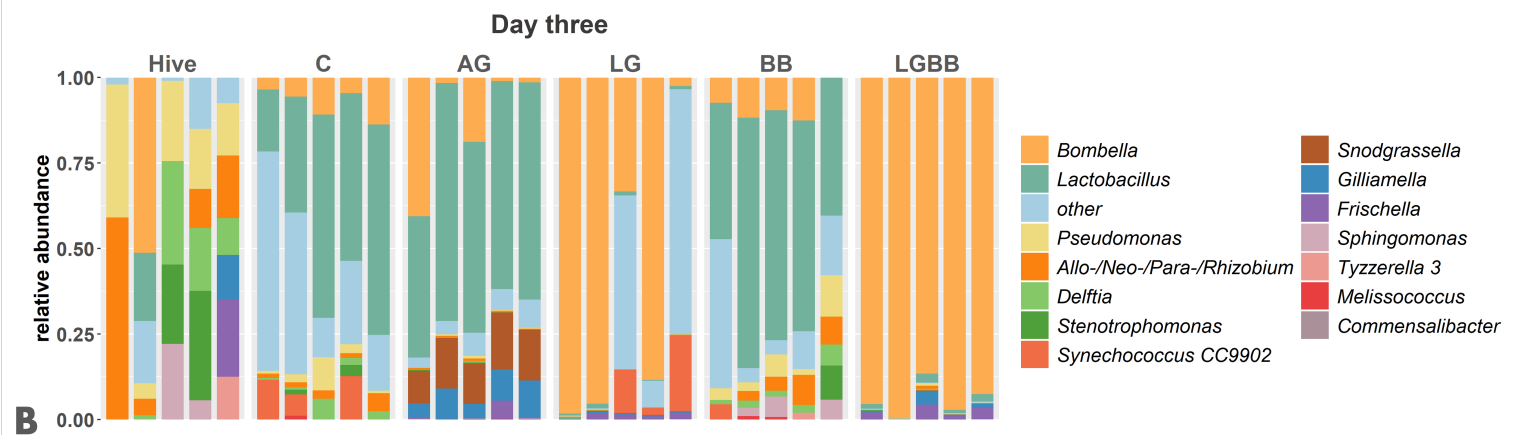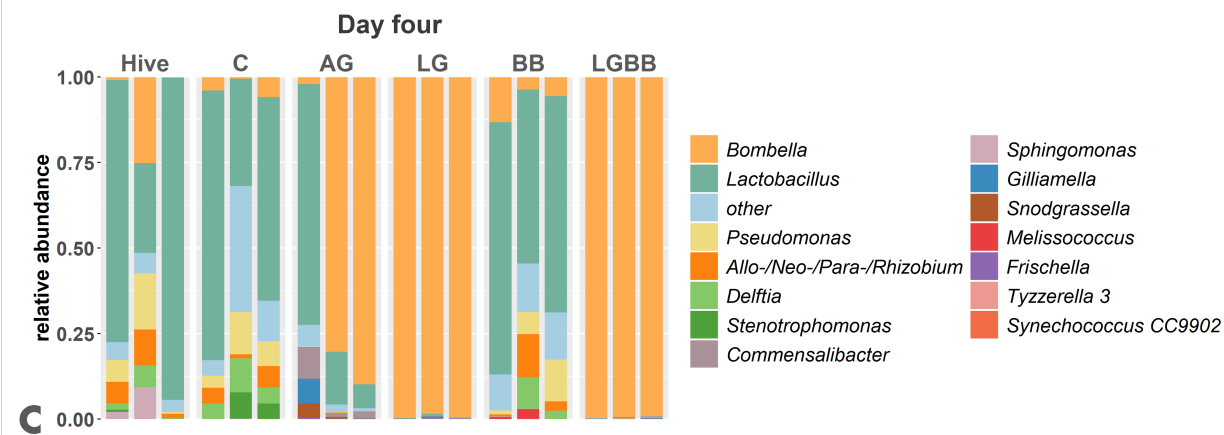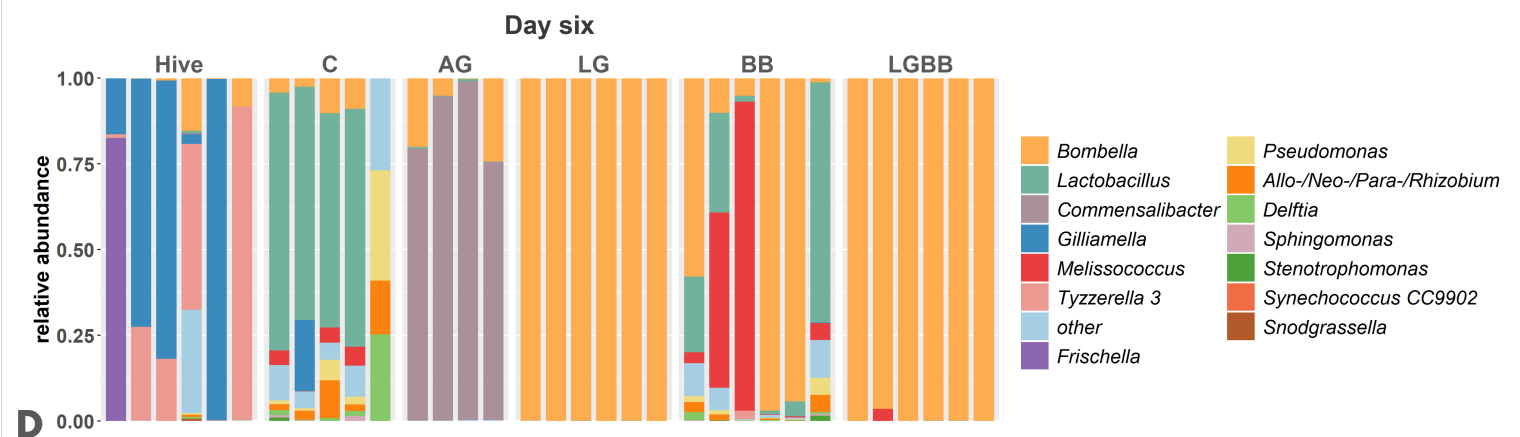

Supplement: FIG S5 [file mbio.02966-21-sf005.pdf]

Anova,  $F(5,23) = 34.2$ ,  $p = <0.0001$ ,  $\eta_g^2 = 0.88$

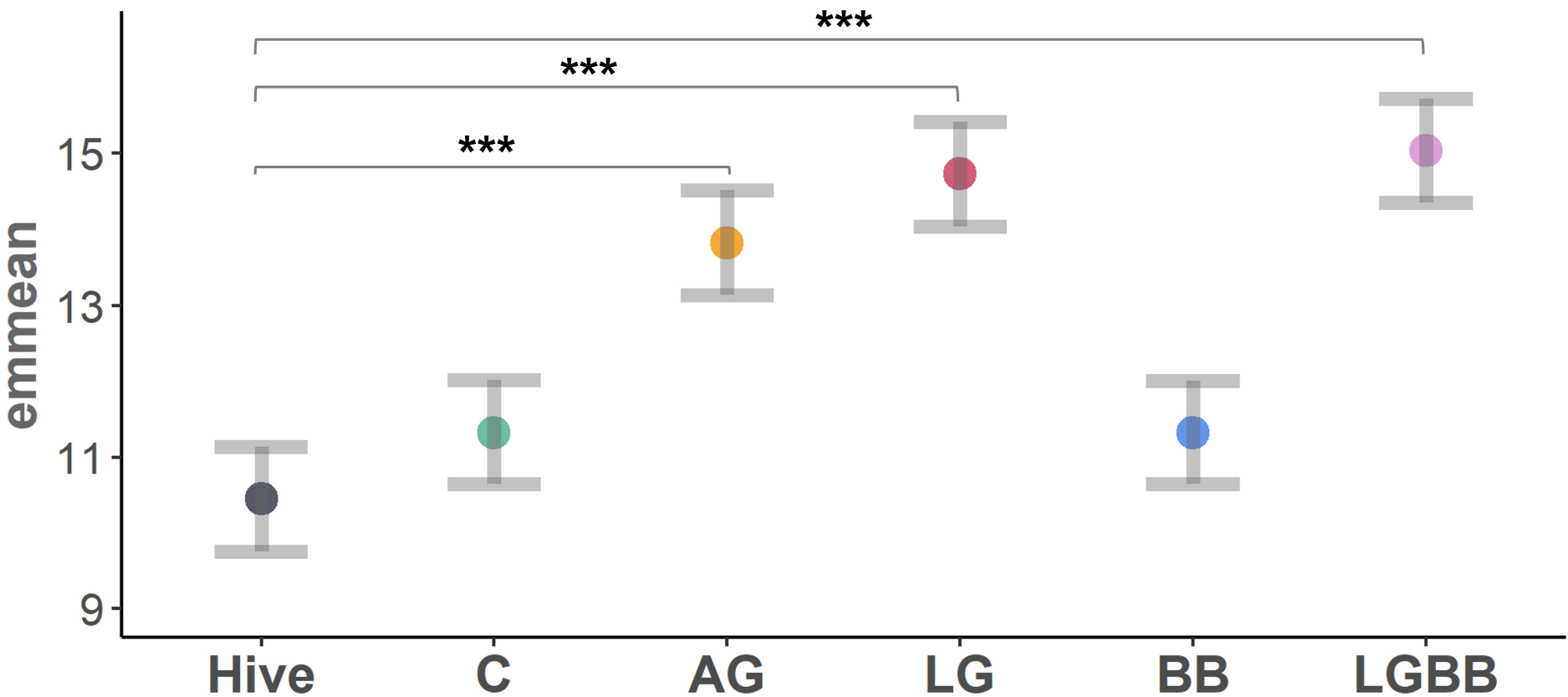

pwc: Emmeans test; p.adjust: Fdr

Supplement: FIG S7 [file mbio.02966-21-sf007.pdf]

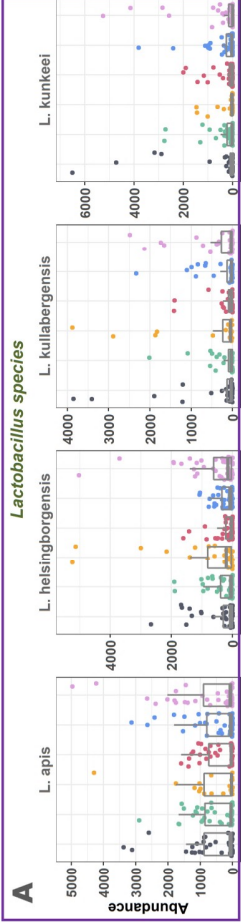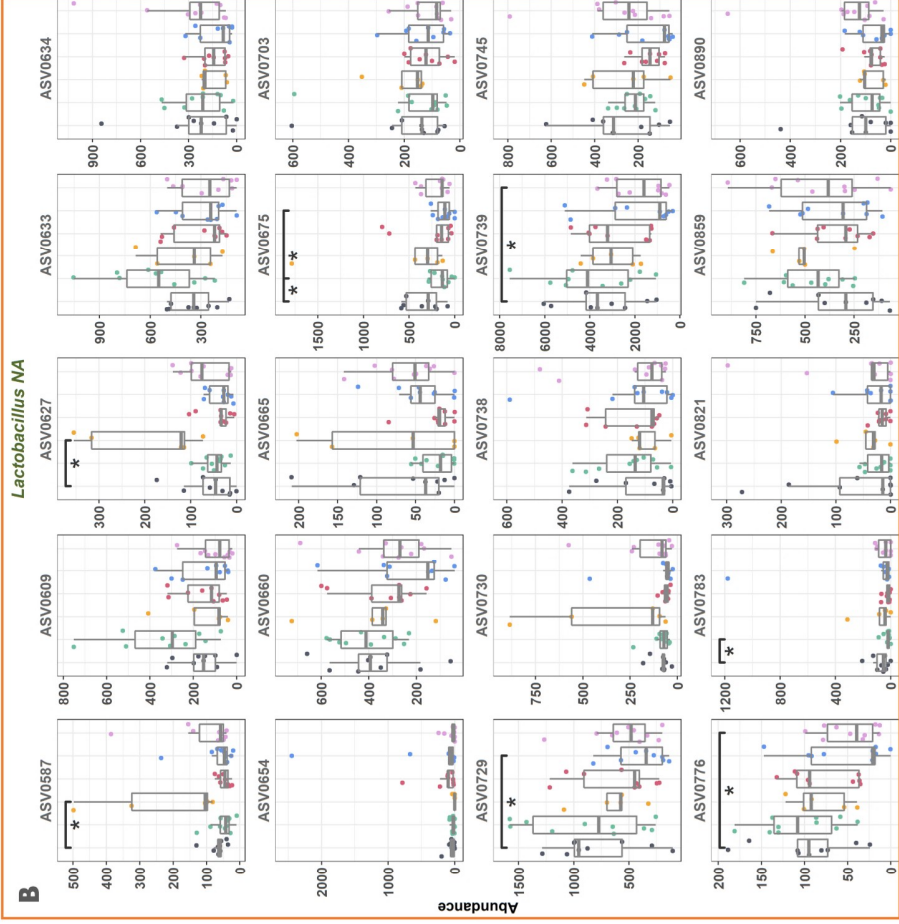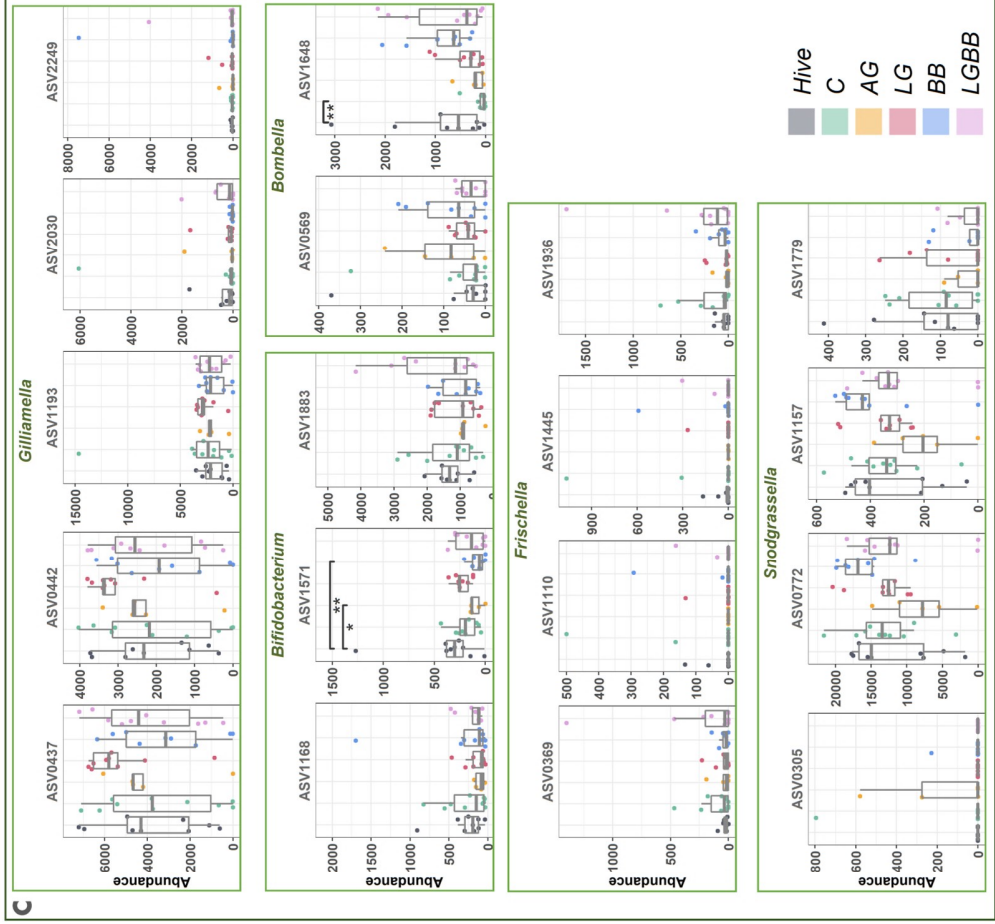

Supplement: FIG S8 [file mbio.02966-21-sf008.pdf]

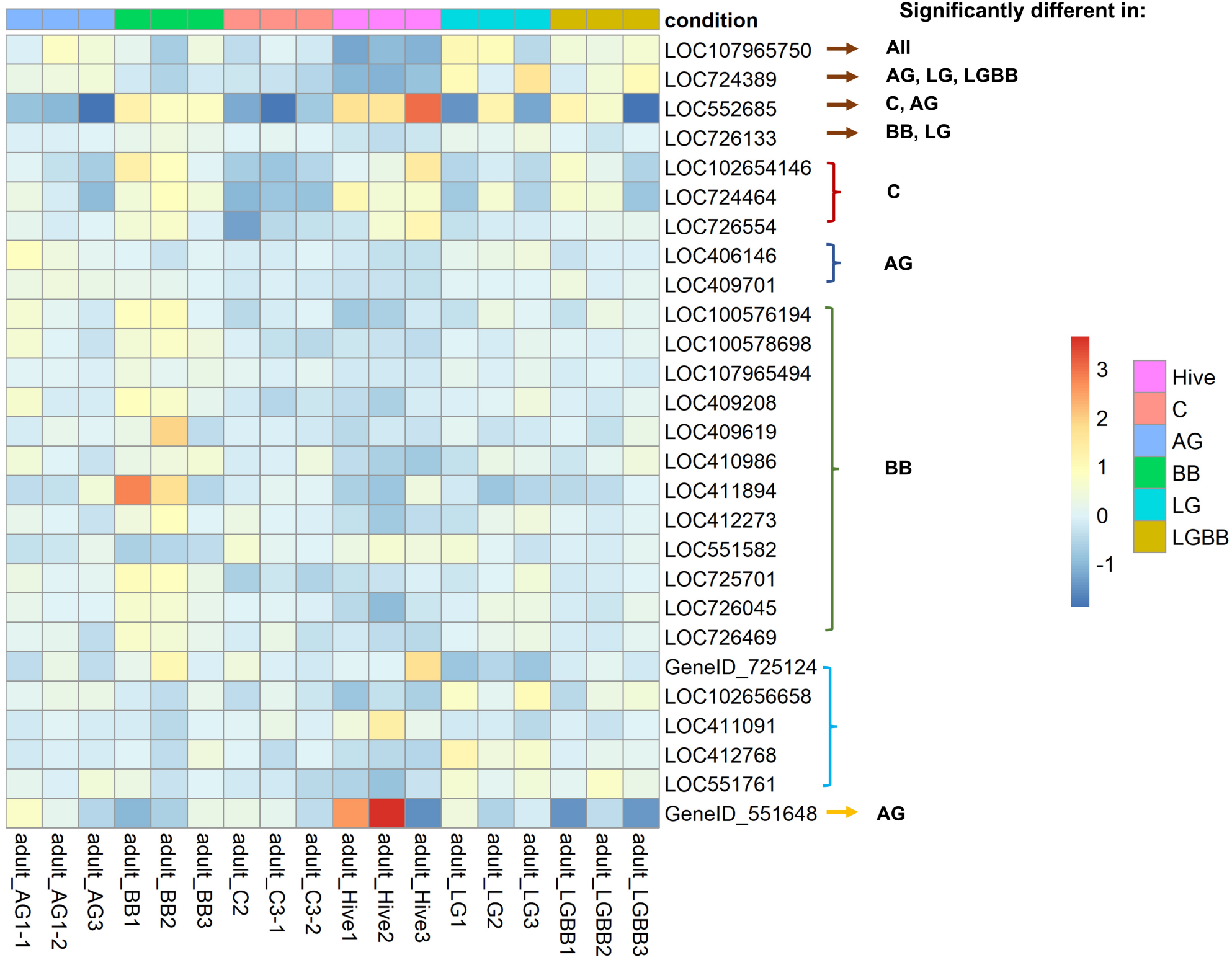

Supplement: FIG S9 [file mbio.02966-21-sf009.pdf]
