# Supplementary material for: Honey Bee Larval and Adult Microbiome Life Stages Are Effectively Decoupled with Vertical Transmission Overcoming Early Life Perturbations
Source: mBio. 2021 Dec 21;12(6):e02966-21. doi: 10.1128/mBio.02966-21 (PMC8689520; doi:10.1128/mBio.02966-21)
Supplement: FIG S4 [file mbio.02966-21-sf004.pdf]

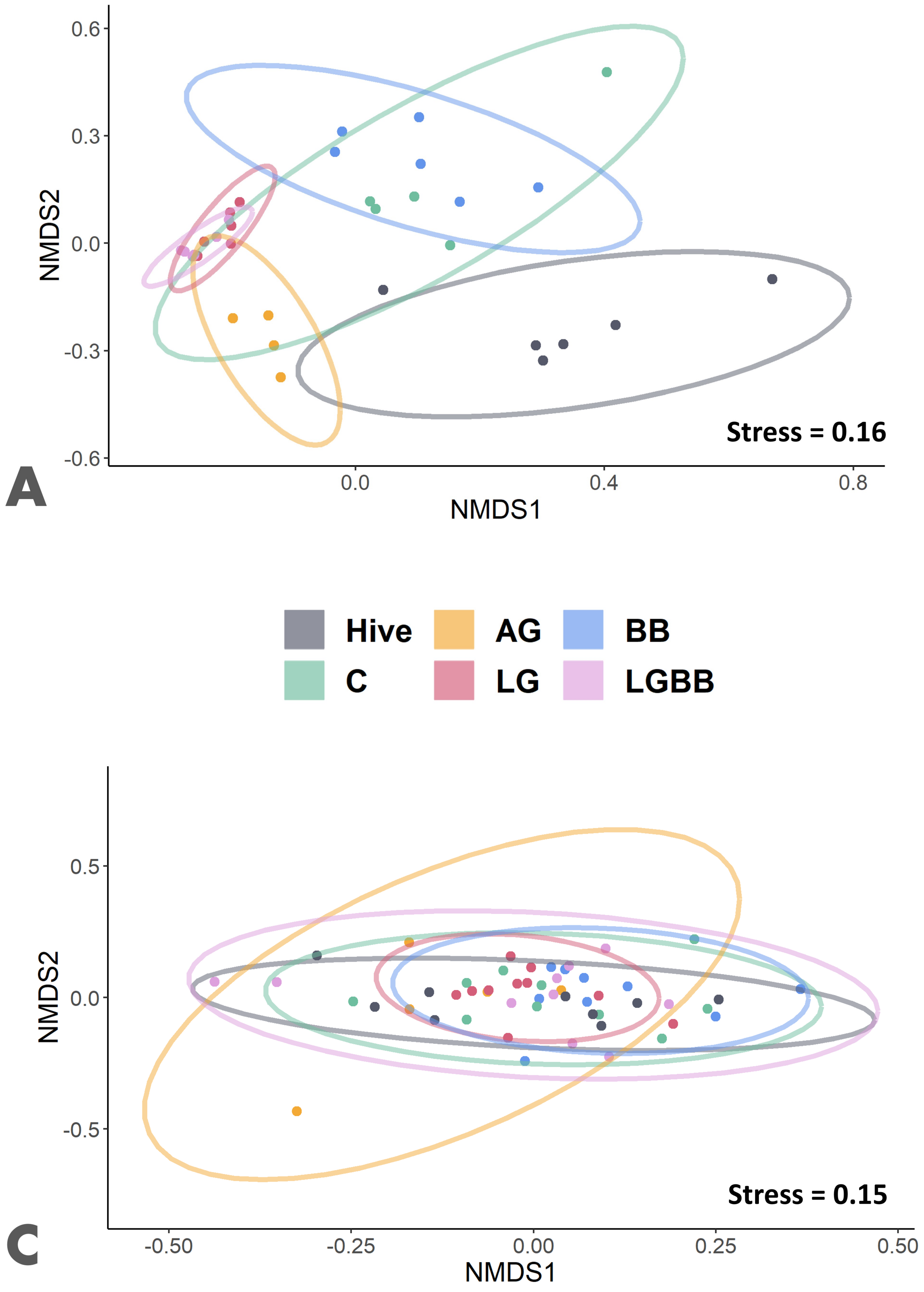

B

time point	treatment	*F*	*R2*	*p*-value	FDR *p*-value
three	C	3.9	0.3	0.01	0.012
three	AG	7.6	0.5	0.006	0.012
three	LG	5	0.4	0.006	0.012
three	BB	4.8	0.4	0.011	0.012
three	LGBB	8.4	0.5	0.012	0.012
four	C	1.7	0.3	0.1	0.2
four	AG	4.9	0.6	0.1	0.125
four	LG	14	0.8	0.1	0.125
four	BB	4.5	0.3	0.002	0.01
four	LGBB	9.1	0.7	0.1	0.125
six	C	4.6	0.3	0.004	0.005
six	AG	7.5	0.5	0.009	0.009
six	LG	10.7	0.5	0.004	0.005
six	BB	4.5	0.3	0.003	0.005
six	LGBB	11.3	0.5	0.003	0.005

D

treatment	*F*	*R2*	*p*-value	FDR *p*-value
C	0.9	0.05	0.42	0.59
AG	1.2	0.09	0.3	0.59
LG	0.9	0.05	0.47	0.59
BB	1.8	0.10	0.11	0.55
LGBB	0.7	0.04	0.62	0.62
