# Supplementary material for: Honey Bee Larval and Adult Microbiome Life Stages Are Effectively Decoupled with Vertical Transmission Overcoming Early Life Perturbations
Source: mBio. 2021 Dec 21;12(6):e02966-21. doi: 10.1128/mBio.02966-21 (PMC8689520; doi:10.1128/mBio.02966-21)
Supplement: FIG S6 [file mbio.02966-21-sf006.pdf]

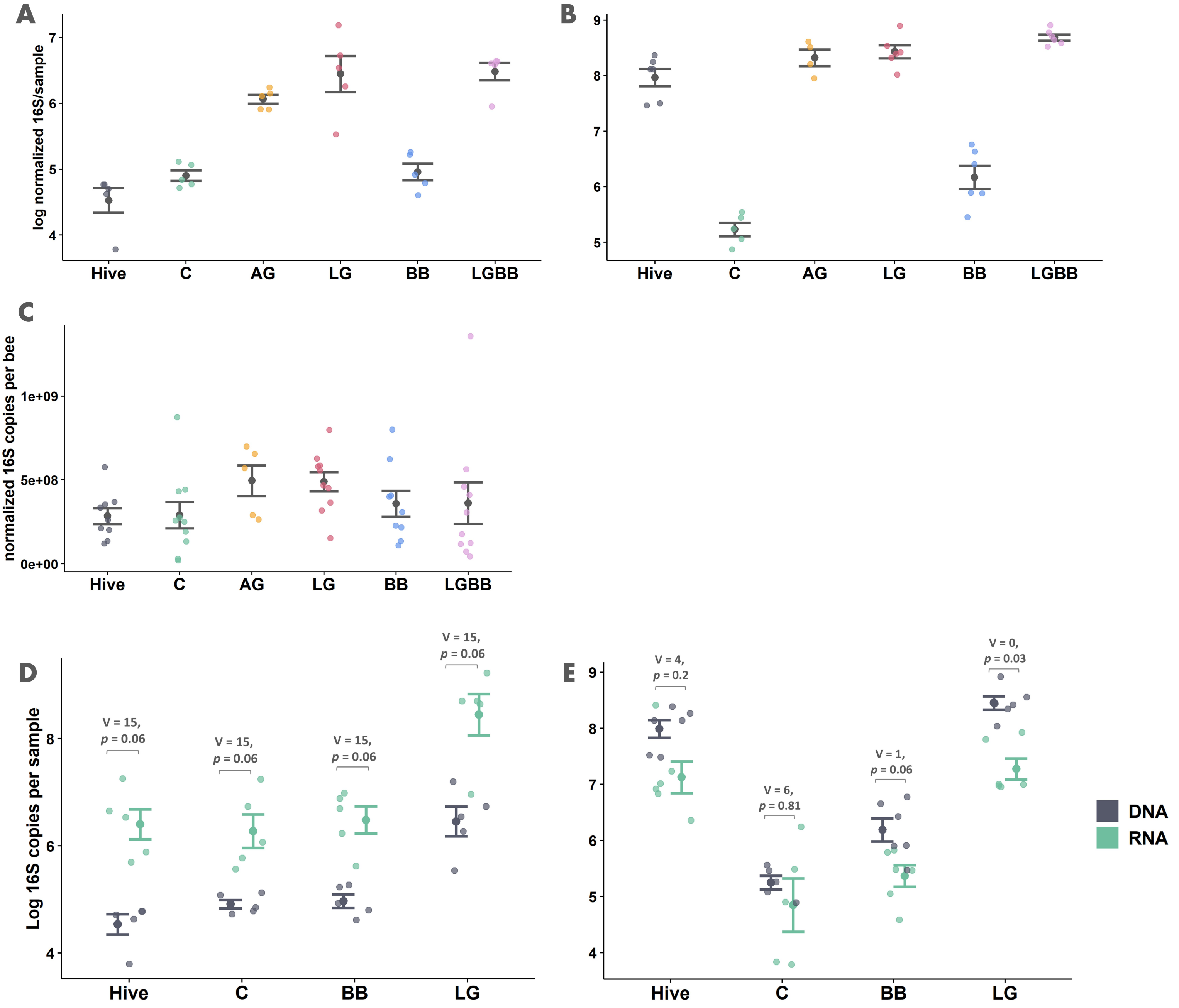

F

Treatment	Time point	samples	DNA as qPCR template			RNA as qPCR template		
			average	min	max	average	min	max
	start larvae 24h	4	2.49E+04	7.44E+03	3.11E+04			
Hive	day three larvae	5	4.30E+04	6.06E+03	5.87E+04	5.40E+06	4.94E+05	1.79E+07
C	day three larvae	5	8.52E+04	5.20E+04	1.30E+05	4.97E+06	3.67E+05	1.74E+07
AG	day three larvae	5	1.21E+06	8.08E+05	1.74E+06			
BB	day three larvae	5	1.06E+05	4.03E+04	1.82E+05	4.89E+06	4.15E+05	9.67E+06
LG	day three larvae	5	5.22E+06	3.37E+05	1.52E+07	6.28E+08	9.15E+06	1.68E+09
LGBB	day three larvae	5	3.47E+06	8.89E+05	4.37E+06			
Hive	day six larvae	6	1.21E+08	2.89E+07	2.31E+08	5.06E+07	2.27E+06	2.59E+08
C	day six larvae	5	1.97E+05	7.43E+04	3.45E+05	4.28E+05	6.11E+03	1.74E+06
AG	day six larvae	4	2.47E+08	8.97E+07	4.12E+08			
BB	day six larvae	6	2.39E+06	2.82E+05	5.71E+06	3.38E+05	3.86E+04	6.72E+05
LG	day six larvae	6	3.27E+08	1.04E+08	7.91E+08	3.10E+07	8.97E+06	8.44E+07
LGBB	day six larvae	6	5.10E+08	3.32E+08	8.05E+08			
Hive	adults	9	2.84E+08	1.20E+08	5.76E+08			
C	adults	9	2.90E+08	1.96E+07	8.73E+08			
AG	adults	5	4.96E+08	2.64E+08	6.99E+08			
BB	adults	9	3.58E+08	1.09E+08	7.99E+08			
LG	adults	10	4.90E+08	1.51E+08	7.97E+08			
LGBB	adults	10	3.62E+08	4.31E+07	1.36E+09			
Hive_nurse	adults	4	4.29E+08	1.29E+08	6.17E+08			
